# Low Dietary Fiber Intake Links Development of Obesity and Lupus Pathogenesis

**DOI:** 10.3389/fimmu.2021.696810

**Published:** 2021-07-15

**Authors:** Anna-Lena Schäfer, Alexandra Eichhorst, Carolin Hentze, Antoine N. Kraemer, Anaïs Amend, Dalina T. L. Sprenger, Cara Fluhr, Stephanie Finzel, Christoph Daniel, Ulrich Salzer, Marta Rizzi, Reinhard E. Voll, Nina Chevalier

**Affiliations:** ^1^ Department of Rheumatology and Clinical Immunology, University Medical Centre Freiburg, Freiburg, Germany; ^2^ Department of Nephropathology, Friedrich-Alexander University (FAU) of Erlangen-Nuremberg, Erlangen, Germany

**Keywords:** autoimmunity, lupus, SLE, diet, fiber, obesity, SCFA

## Abstract

Changed dietary habits in Western countries such as reduced fiber intake represent an important lifestyle factor contributing to the increase in inflammatory immune-mediated diseases. The mode of action of beneficial fiber effects is not fully elucidated, but short-chain fatty acids (SCFA) and gut microbiota have been implicated. The aim of this study was to explore the impact of dietary fiber on lupus pathology and to understand underlying mechanisms. Here, we show that in lupus-prone NZB/WF1 mice low fiber intake deteriorates disease progression reflected in accelerated mortality, autoantibody production and immune dysregulation. In contrast to our original assumption, microbiota suppression by antibiotics or direct SCFA feeding did not influence the course of lupus-like disease. Mechanistically, our data rather indicate that in low fiber-fed mice, an increase in white adipose tissue mass, fat-inflammation and a disrupted intestinal homeostasis go along with systemic, low-grade inflammation driving autoimmunity. The links between obesity, intestinal leakage and low-grade inflammation were confirmed in human samples, while adaptive immune activation predominantly correlated with lupus activity. We further propose that an accelerated gastro-intestinal passage along with energy dilution underlies fiber-mediated weight regulation. Thus, our data highlight the often-overlooked effects of dietary fiber on energy homeostasis and obesity prevention. Further, they provide insight into how intricately the pathologies of inflammatory immune-mediated conditions, such as obesity and autoimmunity, might be interlinked, possibly sharing common pathways.

## Introduction

The significant increase of inflammatory, immune-mediated diseases in Western societies represents a global public health problem. Disorders belonging to this group include cardiovascular disease, the metabolic syndrome, allergies, cancer, and autoimmune diseases. These complex disorders are characterized by chronic inflammation in different organs usually associated with systemic effects. Although each disease has unique epidemiology and pathophysiology, they share the dysregulation of common inflammatory and regulatory immune pathways, central to their pathogenesis (1).

Systemic lupus erythematosus (SLE) is a prototypic autoimmune disease going along with systemic inflammation and severe organ damage, such as nephritis, hematologic or neurological complications. SLE is characterized by a loss of self-tolerance, a broad dysregulation of innate and adaptive immunity and production of autoantibodies against nuclear self-antigens. Although the exact mechanisms are still unresolved, it becomes increasingly clear that the pathogenesis of SLE and other chronic inflammatory and autoimmune diseases results from a complex interplay between genetic and environmental factors. These include infectious agents, toxins as well as various lifestyle and socio-economic factors (2, 3).

Changed dietary habits represent one of the most influential lifestyle factors that have changed significantly over the past few decades. Generally, Westernized diets are characterized by an increased caloric intake and changed nutrient composition. Especially, intake of plant-based fiber has declined below the recommended daily dose (4, 5). Dietary fiber provides health benefits on different levels. Locally, an impact on composition and biodiversity of the microbiome along with an improved intestinal homeostasis and barrier function was reported (6, 7). For instance, populations consuming high amounts of microbiota-accessible carbohydrates display a significant enrichment of *Bacteroides*. In contrast, *Firmicutes* enrichment and *Bacteroides* depletion were found in populations consuming a Westernized diet and are also associated with obesity (8–11). Further advantageous fiber effects on intestinal homeostasis and integrity comprise the promotion of mucus secretion, changed expression of tight-junction proteins and increased IgA secretion by intestinal B cells (7, 12, 13). Moreover, dietary fiber promotes regulatory T cell (T_reg_) expansion (14, 15) and IL-18 production, a cytokine important for epithelial repair (6). Systemically, fiber intake can affect diverse tissues and cells (16) and regulate energy expenditure, appetite and weight *via* neural and humoral pathways (17–19). Hence, in many inflammatory disorders, a fiber-rich nutrition ameliorates inflammation and delays development or progression of disease (20–25). Mechanistically, many studies highlight an important role for dietary-related metabolites controlling the different pathways. A major metabolic product of dietary fiber are short-chain fatty acids (SCFA) that derive from fermentation by commensal bacteria in the gut and modulate cell functions by epigenetic, metabolic or G-protein coupled receptor (GPCR) effects (4).

Here, we show that low compared to high fiber intake accelerates the progression of lupus and associated immune-dysregulation. In contrast to our original assumption, SCFA did not play a pivotal role in mediating beneficial fiber effects. Rather, fiber intake impacted energy homeostasis preventing obesity, intestinal leakage and systemic inflammation along with slower disease progression. Hence, our findings indicate intricate actions of dietary fiber on different immune-mediated processes and disease states that may be interlinked and affect each other.

## Materials and Methods

### Mice and Models

Lupus-prone NZB/WF1 mice were generated by crossing *NZB*/BlNJ with *NZW/LacJ mice.* These and *MRL/MpJ-Faslpr/J* were purchased from *The Jackson Laboratory.* For all experiments, female mice were housed on a 12-h light/dark cycle, with food and *water ad libitum*. To test the influence of dietary fiber, mice were fed different purified diets: a low fiber (LF) diet (Provimi Kliba diet 2122 containing 0.2% cellulose; energy content 12.9 KJ/g), a normal fiber (NF) diet (Provimi Kliba diet 2122 containing 4.7% cellulose; energy content 12.4 KJ/g) and a high fiber (HF) diet (Provimi Kliba diet 2122 containing 4.7% cellulose and 15% pectin (*Herbstreith & Fox*); energy content 11.4 KJ/g). All diets were purchased from *Kliba Nafag*. Provimi Kliba diet 2122 is composed of the following major nutrients: 93.9% dry matter, 18% crude protein, 5% crude fat, 0.3% crude fiber, 3.5% crude ash, 67.1% NFE and 42.5% starch). To test the influence of SCFA, mice were fed standard chow (Provimi Kliba diet 3807.PX.L10), and a mix of 100 mM Na-Acetate, 50 mM Na-Propionate and 50 mM Na-Butyrate (*Sigma-Aldrich*) was supplemented in the drinking water. For gut microbiota suppression, mice were administered a broad-spectrum antibiotic mix of neomycin (1 g/L, *Fisher Scientific)* and vancomycin (0.5 g/L, *Hikma*). All treatments were started after weaning at roughly 4 weeks of age and continued throughout the entire duration of the experiment. To test systemic effects of inflammatory cytokines, mice were injected three times per week and over a period of 6 weeks (20 w–26 w of age) with a mix of recombinant TNFα, IL-6 and IL-1β (100 ng each) or PBS (vehicle). Blood and urine were collected and mice were euthanized at defined time points for organ harvest and downstream experiments. In survival studies, mice were regularly monitored and euthanized when reaching defined ethical endpoints (proteinuria plus deteriorating general health condition and/or significant weight loss). To determine gross food intake, three to five non-nephritic mice of equal age and treatment group were group-housed. On three following days, 24 h food intake was determined as the difference in weight between the food put into that cage and that remaining at the end of 24 h. The mean of the three measurements was calculated and divided by the number of co-housed animals to determine the average intake per mouse over 24 h.

### Human Subjects

**Supplementary Tables 3, 4** give a summary on patients and healthy controls included into the study, their sex, median age (years), SLEDAI, BMI (kg/m^2^), serum levels of leptin (pg/ml), CRP (mg/l), and endotoxemia (pg/ml), and a summary on current treatment, including steroids (prednisone).

### Assessment of Proteinuria

Urine samples were collected by spontaneous urination or in metabolic cages (*Tecniplast*). For a semi-quantitative measurement of proteinuria, Albustix test strips (*Siemens*) were used. According to the color scale provided by the manufacturer, albuminuria was categorized as follows: 0–1 = trace, 1 = 30, 2 = 100, 3 = 300 and 4 >2,000 mg/dl.

### Assessment of Anti-dsDNA Autoantibodies and IgG Subclasses

IgG, IgG2a and IgG1 antibody secretion directed against dsDNA was determined by enzyme-linked immunosorbent assay (ELISA). Briefly, 384-well microtiter plates (*Greiner Bio One*) were pre-coated with 20 µg/ml Poly-L-Lysin (*Sigma-Aldrich*) for 1 h at 37°C followed by coating with 20 µg/ml calf thymus DNA (*Sigma-Aldrich*) at 4°C o.n. Plates were blocked with 2% fetal calf serum (FCS) in PBS for 2 h at RT. Samples were diluted in 2%FCS in PBS and incubated for 2 h at RT. Bound anti-dsDNA immunoglobulins were detected with HRP-conjugated secondary antibodies specific for mouse IgG, IgG1 or IgG2a (*SouthernBiotech*), followed by development with TMB substrate (*Thermo Fisher Scientific)* according to the manufacturer’s protocol. The absorbance at 450 nm was measured using the Spark^®^ 10 M multimode microplate reader (*Tecan*). To determine autoantibody titers, expressed as arbitrary unit (A.U.), reference sera were used to create a standard curve.

### Flow Cytometry

#### Mouse Samples

Single cell suspensions of spleen and kidney were obtained by mechanic dissociation [in some experiments by enzymatic digestion using collagenase (5 mg/ml, *Life Technologies*)]. Following incubation with anti-CD16/32 antibodies (101330*, BioLegend*) to block non-specific Fc receptor binding, single cell suspensions were stained with biotin- or fluorochrome-conjugated monoclonal antibodies diluted in 2% FCS/PBS for 30 min on ice. For intracellular or intranuclear staining, cells were fixed and permeabilized with BD Cytofix/Cytoperm *(BD Biosciences*) or eBioscience FoxP3/Transcription Factor Staining Buffer Set (*eBioscience*), respectively. For intracellular cytokine staining, cells were re-stimulated with 50 ng/ml PMA (*Sigma-Aldrich*), 1 µg/ml Ionomycin (*Sigma-Aldrich*) and Brefeldin A (*eBioscience*) for 4 h at 37°C/5%CO_2_ prior staining and fixation. The following antibodies were used: Annexin V APC (*640941*, *BioLegend*), TCR β chain Biotin (*109203, BioLegend*), CD19 Biotin (*115505, BioLegend*), NK1.1 Biotin (*108704, BioLegend*), Ly6G Biotin (*127604, BioLegend*), CXCR5 Biotin (*551960, BD Biosciences*), κ light chain Biotin (*559750, BD Biosciences*), λ light chain Biotin (*553433, BD Biosciences*), CD11c APC (*170111482, eBioscience*), CD11b FITC (*101205, BioLegend*), Ly6G V450 (*560603, BD Biosciences*), Ly6C PE-Cy7 (*560593, BD Biosciences*), CD45 APC-Cy7 (*103115, BioLegend*), PDCA1 PE (*1231782, eBioscience*), Streptavidin-PerCP-Cy5.5 (*45431782, BD Biosciences*), CD45R/B220 Pacific Blue (*103230, BioLegend*), CD11c PE-Cy7 (*117318, BioLegend*), CD80 APC-Fire750 (*104738, BioLegend*), CD86 APC (*7086281, eBioscience*), TCR β chain APC-Cy7 (*109219, BioLegend*), CD4 PE-Cy7 (*100422, BioLegend*), CD8a PerCP (*100732, BioLegend*), PD-1 PE (*1299858, eBioscience*), Streptavidin-APC (*17431782, eBioscience*), CD44 FITC (*103021, BD Biosciences*), CD45 eFluor506 (*69045182, eBioscience*), IFNγ APC (*505809, BioLegend*), IL-10 FITC (*505005, BioLegend*), IL-17 PE (*559302, BD Biosciences*), FoxP3 APC (*17577382, eBioscience*), CD103 PE (*557495, BD Biosciences*), CD138 PE (*5537, BD Biosciences*), CD45R/B220 APC-Cy7 (*103224, BioLegend*), Streptavidin-V450 (*560797, BD Biosciences*), GL7 FITC (*553666, BD Biosciences*), Fas PE (*554258, BD Biosciences*), TCRβ chain PerCP (*109227, BioLegend*), CD21 FITC (*553818, BD Biosciences*), and CD23 PE (*553139, BD Biosciences*). The following immune cell subsets were identified (**Supplementary Figures 5, 6**): B cells (% TCRβ^-^B220^+^/live or CD45^+^ cells), germinal center B cells (% Fas^hi^GL7^hi^/B cells), plasma cells/blasts (% CD138^hi^LC^+^/live cells), marginal zone B cells (% CD21^hi^CD23^lo^/B cells), follicular B cells (% CD21^lo^CD23^hi^/B cells), CD4^+^ and CD8^+^ T cells (%TCRβ^+^B220^−^CD4^−^CD8^+^ or TCRβ^+^B220^−^CD8^−^CD4^+^/live or CD45^+^ cells), expression of IFNγ, IL-17, IL-10 and CD44^hi^ on CD4^+^ or CD8^+^ T cells, regulatory T cells (T_reg_) (% FoxP3^+^/CD4^+^ T cells), effector T_reg_ (% CD103^+^/T_reg_), follicular T helper cells (T_FH_) (% CXCR5^hi^PD-1^hi^/CD4^+^ T cells), cDC and pDC (CD11c^hi^ or PDCA1^hi^ CD19^−^TCRβ^−^NK1.1^−^/live or CD45^+^ cells), neutrophils (Ly6G^hi^CD11b^+^CD19^−^TCRβ^−^NK1.1^−^/live or CD45^+^ cells), CD11b^+^ monocytic cells (Ly6G^-^CD11b^+^CD19^−^TCRβ^−^NK1.1^−^/live or CD45^+^ cells) and expression of CD80 or CD86 on monocytic cells and cDC. In kidney, additionally CD45 staining was included. To identify apoptotic cells, Annexin V staining was performed using Annexin V Binding Buffer (*BD Biosciences*).

#### Human Samples

Whole EDTA-blood was stained with fluorochrome-conjugated monoclonal antibodies followed by red blood cell lysis using OptiLysis B (*Beckman Coulter*) according to manufacturer’s protocol. The following antibodies were used: CD3 BV421 (562436, *BD Biosciences*), CD4 PE-Cy7 (348809, *BD Biosciences*), CD8 PerCP (344707, *BioLegend*), HLA-DR FITC (347400, *BD Biosciences*). Determined were: expression of the activation marker HLA-DR on whole CD3^+^, CD3^+^CD4^+^ or CD3^+^CD8^+^ T cells.

Flow cytometric analysis was performed at BD LSR Fortessa flow cytometer (*Becton Dickinson*) followed by data analysis using FlowJo™ Software (*Becton Dickinson*).

### Real-Time Quantitative PCR

Total RNA was extracted using TRIzol reagent (*Invitrogen*), and the QuantiTect Reverse Transcription Kit (*Qiagen*) was used for cDNA synthesis according to the manufacturer’s instructions. Transcripts were quantified by real-time quantitative PCR on a StepOnePlus™ Real-Time PCR System (*Applied Biosystems*) with predesigned TaqMan Gene Expression Assays and reagents according to manufacturer’s instructions (*Applied Biosystems*). Probes with the following Applied Biosystems assay identification numbers were used: Mm99999915_g1 (*GAPDH*), Mm00434226_m1 (*IL-18*), Mm00434228_m1 (*IL-1β*), Mm00446190_m1 (*IL-6*), Mm00434759_m1 (*Leptin*), Mm01276696_m1 (*Muc2*), Mm00441127_m1 (*Reg3*), Mm00443258_m1 (*TNFα*) and Mm00500912_m1 (*ZO-1*). For each sample, *mRNA* abundance was normalized to the amount of GAPDH and is presented in arbitrary units (A.U.).

### Assessment of Intestinal Integrity by FITC Dextran Assay

To measure intestinal leakage, FITC-Dextran (*Sigma-Aldrich*) was administered *via* oral gavage (600 mg/kg body weight). After 4 h, mice were sacrificed and blood was collected for serum preparation. Fluorescence intensity (excitation 485 nm, emission 535 nm) was measured in 96-well black flat-bottom plates (*Corning™Costar™*, *Thermo Fisher Scientific*) at the Spark^®^ 10M multimode microplate reader (*Tecan*).

### Assessment of Endotoxemia by LPS Measurement

Serum LPS levels were measured using HEK-Blue LPS Detection assay (*Invivogen*). HEK-Blue™ hTLR4 cells were cultured in DMEM high glucose medium (*Gibco*) supplemented with GlutaMAX (*Gibco*), 1% Penicillin/Streptomycin (*Gibco*) and HEK-Blue Selection (*Invivogen*) at 37°C in humidified air supplemented with 5% CO_2_. To measure LPS in serum samples, 2.5 × 10^4^ cells were incubated for 24 h in the presence of 20 µl cell culture medium plus 80 µl serum. Secreted embryonic alkaline phosphatase (SEAP) activity in supernatant was then measured by incubating 180 µl QUANTI Blue Solution with 20 µl cell culture supernatant for 90 min at 37°C. OD at 620 nm was measured at the Infinite F50 microplate reader (*Tecan*). A negative control (culture medium) was included as well as supernatants of cells incubated with different LPS concentrations (*E. coli* O55:B5, *Sigma-Aldrich*) to calculate a standard curve.

### Assessment of Cytokine and Leptin Levels by Plex Assays

Cytokine levels in mouse serum were determined using the LEGENDplex Mouse Inflammation Panel (*BioLegend*), human leptin levels in serum using a customized LEGENDplex Panel (*BioLegend*) and mouse leptin using a customized Bio-Plex assay (*Bio-Rad*). The assays were performed according to the manufactures instructions, LEGENDplex assays were analyzed at BD LSRFortessa flow cytometer (*Becton Dickinson*), Bio-Plex assays at Luminex LX200 (*Bio-Rad*).

### Statistics

For statistical analysis Instat software Prism (*GraphPad software*) was used. *P*-values less than or equal to 0.05 were considered significant. Statistical comparison between two, or more than two experimental groups were performed using Mann–Whitney U-test and Kruskal–Wallis-test, respectively. The Kaplan–Meier method was used for estimating and displaying OS rates. Outliers were determined by ROUT method. To analyze the association between different parameters Spearman’s rank correlation coefficients were calculated.

### Study approval

#### Animal

Animal experiments were approved by the local governmental commission for animal protection of Freiburg (Regierungspräsidium Freiburg, approval nos. G16/58 and G19/21).

#### Human

Experiments were conducted according to the principles expressed in the Declaration of Helsinki as Ethics Statement. The study was in accordance with the ethical standards set by the institutional ethics committee (RB approval numbers 210/20 and 624/14). All participants gave their informed consent prior to the inclusion in the study.

## Results

### Low Intake of Dietary Fiber Deteriorates Disease Progression in Lupus-Prone NZB/WF1 Animals

**Figure 1:**
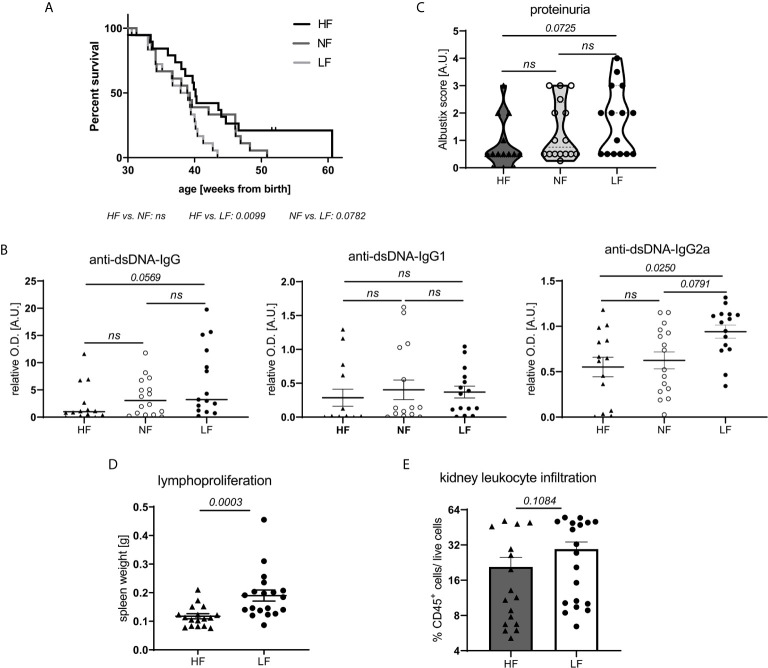
Low intake of dietary fiber accelerates disease in lupus-prone NZB/WF1 mice. **(A)** Overall survival (OS) determined in lupus-prone NZB/WF1 mice fed a high (HF; n = 19), normal (NF; n = 18) or low fiber (LF; n = 18) diet. The Kaplan–Meier method was used for estimating OS in differently treated groups. **(B–D)** Determination of **(B)** total anti-dsDNA-IgG, -IgG1 and -IgG2a serum titers, **(D)** lymphoproliferation by spleen weight, **(C, E)** signs of beginning nephritis by kidney-infiltrating CD45^+^ leukocytes and proteinuria levels, in 28 w old animals fed a HF (n = 14–17 mice), NF (n = 16 mice) or LF (n = 15–19 mice) diet. Results are expressed as scatter blots with mean ± SEM; each data point represents an individual mouse; *p < 0.05* was considered significant, *p > 0.2* is indicated as *ns, not significant*.

To explore the effects of fiber intake on lupus pathogenesis, lupus-prone NZB/WF1 mice were continuously fed a high (HF), normal (NF) and low fiber (LF) diet. In addition to poorly fermentable cellulose, the soluble fiber pectin was supplemented in HF diet as this had resulted in markedly diverse gut microbial communities and high intestinal SCFA levels in previous studies (25). Considering an average intake of 8,370 KJ per day, the consumption of a HF, LF or NF diet would correspond to an intake of 144, 1.29 or 31.75 g fiber per day, respectively. Hence, the fiber content of the NF diet corresponds roughly to the recommended daily fiber intake of human adults, while HF and LF diet are at the upper and lower end as in many other studies. Generally, LF-treated mice showed accelerated disease progression compared to HF-treated mice, reflected in significantly reduced overall survival (OS) (**Figure 1A**). Compared to NF-fed mice, OS also appeared to be reduced, however this was not statistically significant. Overall and in accordance with that, we detected highest serum levels of anti-dsDNA-IgG in the LF-cohort. Serum levels of anti-dsDNA-IgG2a, an IgG subclass known to mediate pro-inflammatory effector function (26), were found to be significantly increased in the LF- compared to the HF-group (**Figure 1B**). Compared to NF-treated mice, anti-dsDNA-IgG2a levels were only slightly and not significantly increased, as observed for OS. No differences were found for anti-dsDNA-IgG1, while whole anti-dsDNA-IgG was slightly, although not significantly increased in LF- compared to HF-treated mice (**Figure 1B**). Given these mild, dose-dependent effects of dietary fiber on lupus progression, all further experiments to clarify the underlying pathology were performed with HF- versus LF-treatment only. More advanced lupus-like disease in LF-treated animals was further reflected in a significantly higher lymphoid hyperplasia/splenomegaly (**Figure 1D**), but only slightly increased proteinuria and frequencies of kidney-infiltrating leukocytes, indicative of renal injury (**Figures 1C, E**). Likewise, LF-feeding reduced OS in lupus-prone MRL/*lpr* mice, while no significant anti-dsDNA-IgG titer differences were found (**Supplementary Figure 1**).

Altogether, these results indicate overall detrimental effects of low fiber intake on lupus pathogenesis. Nutritional intake of high compared to normal fiber amounts did only yield slight additional benefits.

### Low Intake of Dietary Fiber Accelerates the Adoption of an Inflamed Immune Phenotype

**Figure 2:**
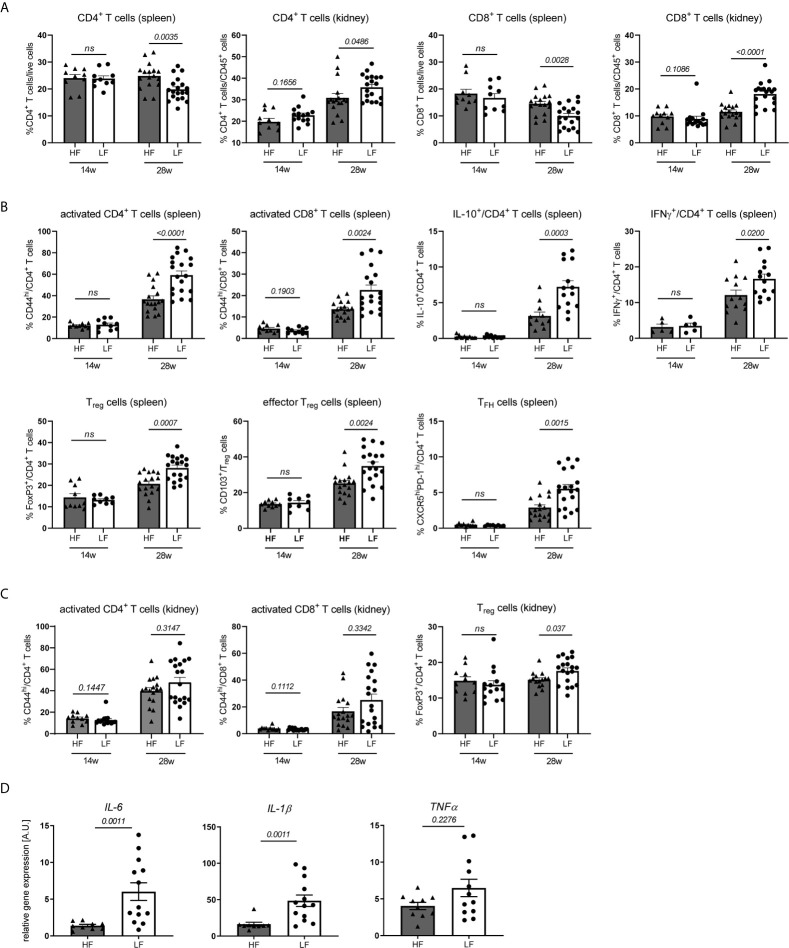
HF-treated mice display a less inflamed immune status. **(A–C)** Broad immune status evaluation in HF- and LF-treated NZB/WF1 mice by FACS in 14 w (n HF/LF = 10–15 mice) and 28 w old animals (n HF = 11–17 mice, n LF = 14–19 mice). **(A)** Relative proportions of CD4^+^ and CD8^+^ T cells in spleen and kidney. **(B, C)** Most prominent differences in immune cell differentiation between HF- and LF-treated mice: CD44^hi^ expression on CD4^+^ and CD8^+^ as marker of T cell activation, expression of IL-10 or IFNγ on CD4^+^ T cells, frequencies of FoxP3^+^ T_reg_, CD103^+^FoxP3^+^ effector T_reg_ and CXCR5^hi^PD-1^hi^ T_FH_ cells. **(D)** Relative *mRNA* expression of IL-6, IL-1β and TNFα was examined in kidneys of 28 w old animals (n HF = 9–10 mice, n LF = 12–13 mice). Results are expressed as scatter blots with mean ± SEM; each data point represents an individual mouse; *p < 0.05* was considered significant, *p > 0.2* is indicated as *ns, not significant*.

Dietary fiber and SCFA are known to have broad effects on innate and adaptive immunity (16) going along with changed disease pathologies. To clarify such a connection in our model we examined systemic changes in distribution, activation and differentiation of major immune cell populations in spleen and kidney of 12–14 w old, yet healthy NZB/WF1 mice, in 28 w old animals with established autoantibodies but no signs of overt nephritis (proteinuria ≤30 mg/dl) and such with signs of advanced disease (proteinuria ≥300 mg/dl) (**Figure 2** and **Supplementary Table 1**).

Expectedly, we noted a significant increase in lymphoproliferation and total cell counts as well as immune phenotypical changes in healthy compared to diseased animals (**Supplementary Figure 2A**; **Supplementary Table 2**). With respect to the distribution of main immune cell populations, most pronounced was the relative decrease of CD8^+^ and to a lesser degree CD4^+^ T cells in spleen and their concomitant accumulation in kidneys. We were not able to clarify the reason underlying these frequency shifts. Spleen T cells in nephritic compared to healthy NZB/WF1 animals did neither show an increased susceptibility to apoptosis, nor was there a more pronounced migration of T cells towards inflamed versus non-inflamed kidneys (**Supplementary Figure 3**) under the employed conditions. Further possible explanations could be an enhanced T cell proliferation in inflamed organs as well as a lower replenishment of the peripheral cell pool by the cytopenic bone marrow (data not shown) of diseased animals. In contrast to kidneys, we found a relative increase of splenic neutrophils and classical dendritic cells (cDC), while plasmacytoid dendritic cells (pDC) dropped in both organs. While expression of co-stimulatory CD80 and CD86 on cDC and CD11b^+^ monocytic cells was not consistently increased, adaptive immune cells adopted an ‘inflamed phenotype’ with progressing disease. In both, kidney and spleen, this comprised a significantly higher expression of the activation marker CD44 and cytokine IFNγ on T cells, while IL-17 was barely expressed, an enhanced T_FH_ differentiation along with increased B cell differentiation in plasma and germinal center (GC) B cells and a concomitant decrease in marginal zone (MZ) and follicular B cells. Strong co-expression of CD44 and IFNγ in CD4 and CD8 T cells (**Supplementary Figure 4**) might point out that activated T cells also represent main mediators of important effector functions. CD4^+^ T cells of diseased animals also displayed elevated levels of anti-inflammatory IL-10, and greater frequencies of regulatory T cells (T_reg_) and CD103^+^ effector T_reg_ in kidney and spleen, which we interpret as counter-regulatory anti-inflammatory immune response in the setting of active disease. Consistent with that, an increase of T_regs_ and IL-10 is reported in lupus-prone mice and patients with disease progression (27).

In accordance with the slower disease progression and less pronounced lymphoproliferation in 28 w old HF- compared to LF-treated mice we not only found reduced splenocyte counts (**Supplementary Figure 2B**), but also immune changes that more closely resembled that of yet healthy animals, predominantly within the T cell compartment (**Supplementary Table 1** and **Figure 2**). Here, we noted not only significant differences in CD4^+^ and CD8^+^ frequency shifts in spleen and kidney (**Figure 2A** and **Supplementary Table 1**), but also an increased expression of CD44, IFNγ and IL-10 on CD4^+^ T cells and higher frequencies of T_reg_, effector T_reg_ and T_FH_ cells in LF-mice (**Figure 2B** and **Supplementary Table 1**). In parts, these patterns of a more inflamed immune phenotype were also found in kidney CD4^+^ (increase in IFNγ^+^ and FoxP3^+^ T_reg_) and spleen CD8^+^ T cells (CD44^hi^ expression), as well as in innate immune cells (CD80 and CD86 expression on cDC, relative drop in spleen and kidney pDC, kidney neutrophils and CD11b^+^ monocytic cells) (**Figures 2B, C** and **Supplementary Table 1**). Supporting these observations, partly similar differences in spleen T cells and innate immune cells were also noted for nephritic HF- versus LF-treated animals, while only sporadic differences were found in the inflamed kidney. Kidneys of 28 w old LF-treated mice also displayed a significantly increased expression of inflammatory *IL-1β* and *IL-6 mRNA* (**Figure 2D**). No pioneering differences were noted in 14 w old, yet healthy animals (**Supplementary Figures 2B, C**; **Supplementary Table 1**).

To summarize, the more favorable disease course in HF- compared to LF-treated animals was also reflected in immunologic changes. Given, that these appeared at large and only at later disease stages, may argue against the notion that dietary fiber skews the differentiation of specific immune cells; it does however not exclude such a scenario. Our results may rather point out that low fiber intake accelerates immune-dysregulation and in concert with that disease progression.

### Intake of Dietary Fiber Impacts Weight Development, Intestinal Homeostasis, and Adipose Tissue and Systemic Inflammation Affecting Disease Pathology

**Figure 3:**
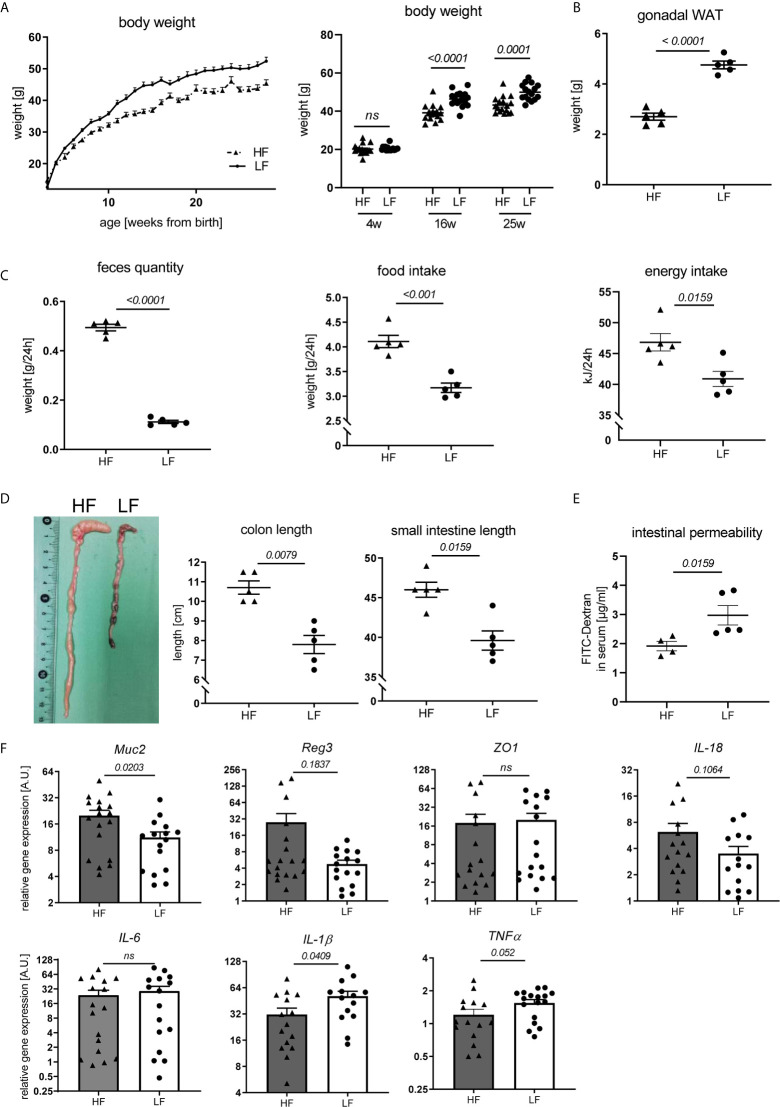
Impact of dietary fiber on weight regulation and intestinal homeostasis. Lupus-prone NZB/WF1 mice were fed a HF- or LF-diet. **(A)** Body weights are shown from 3–28 weeks of age (left) and at defined time points (right) (n HF = 18 mice, n LF = 16 mice; representative of two independent experiments). **(B–D)** Weight of gonadal white adipose tissue (WAT) **(B)**, feces quantity, food and energy intake per day **(C)** as well as lengths of small intestine and colon **(D)** were determined at 28 w (n HF/LF = 5 mice, representative of at least two independent experiments). **(E)** Quantification of intestinal permeability with FITC-Dextran applied by oral gavage in 28 w old animals (n HF = 4 mice, n LF = 5 mice, representative for two independent experiments). **(F)** Relative *mRNA* expression of molecules associated with gut barrier function and inflammation in colon of 28 w old animals (n HF = 15–18 mice, n LF = 15–17 mice). Results are expressed as scatter blots or trend line with mean ± SEM; each data point represents an individual mouse; *p < 0.05* was considered significant, *p > 0.2* is indicated as *ns, not significant*.

Fiber intake can influence disease and immune responses in different ways. Given reported associations between fiber consumption, weight regulation, intestinal homeostasis and autoimmune pathology (28, 29) we explored such a connection in our study. We noted that HF-fed mice continuously presented a lower body weight with the exception of the first weeks after weaning (**Figure 3A**). This was paralleled by a clearly decreased mass of gonadal white adipose tissue (WAT) (**Figure 3B**). Largely increased feces production combined with significantly higher daily chow consumption and energy intake suggest that an accelerated gastro-intestinal passage along with energy dilution may underlie the lower weight gain in these mice (**Figure 3C**). Significantly increased colon and small intestine lengths further support these assumptions (**Figure 3D**). These may result from the hygroscopic pectin effects increasing fecal volume and viscosity, posing a higher mechanical strain and stretch on the intestinal wall and stronger propulsive peristalsis (29, 30). Consequently, fiber intake also affected intestinal homeostasis. In FITC-dextran permeability assays, HF-treated animals showed improved intestinal integrity (**Figure 3E**) and a significant transcriptional upregulation of *Mucin-2*, known to be involved in mucus layer formation and protection of the intestinal wall from self-digestion and microbial contact (31) (**Figure 3F**). We also found a slight, but not significant transcriptional upregulation of molecules associated with antimicrobial defense (*Reg3b*) as well as epithelial repair (*IL-18*), while the gut barrier molecule *ZO-1* was not differently expressed (**Figure 3F**). While IL-6 was equally expressed between LF and HF animals, IL-1β and TNFα showed a significant and slight increase in the colon of LF mice, respectively (**Figure 3F**). In addition to the increased intestinal leakage, this might point towards a slightly enhanced intestinal inflammation.

**Figure 4:**
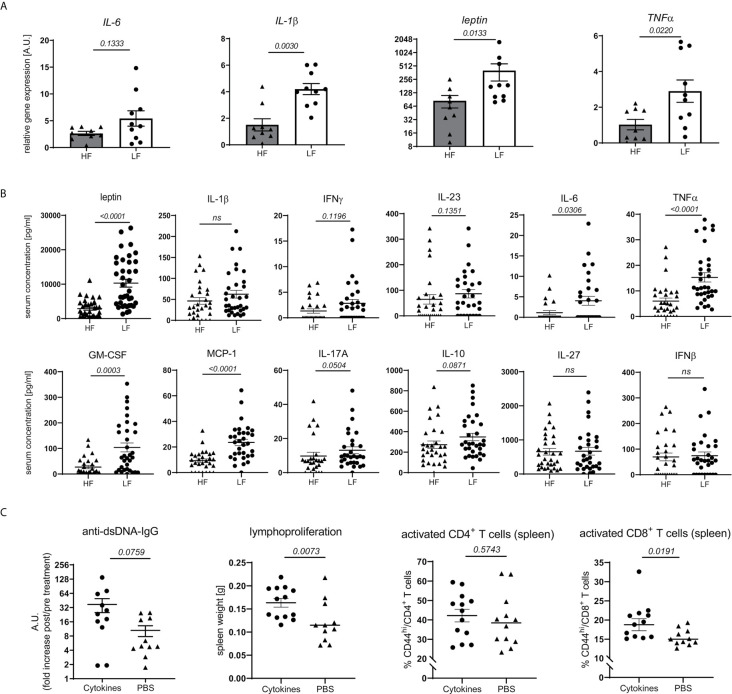
Link between dietary fiber, WAT and systemic inflammation and lupus pathology. Lupus-prone NZB/WF1 mice were fed a HF- or LF-diet. **(A)** Relative *mRNA* expression of IL-1β, IL-6, TNFα and leptin was examined in gonadal WAT of 28 w old animals (n HF = 9 mice, n LF = 10 mice). **(B)** Serum concentrations of leptin and a panel of cytokines in 28 w old animals were determined by Plex assays (n HF = 26–35 mice, n LF = 30–38 mice). **(C)** 20 w old NZB/WF1 mice were treated over a period of 6 weeks with a mix of recombinant TNFα, IL-6 and IL-1β or PBS; determined was the increase in anti-dsDNA-IgG post- compared to pre-treatment as well as development of lymphoproliferation (spleen weight) and expression of CD44 on CD4^+^ and CD8^+^ T cells post-treatment (n cytokines = 11–13; n PBS = 11–12). Results are expressed as scatter blots with mean ± SEM; each data point represents an individual mouse; *p < 0.05* was considered significant, *p > 0.2* is indicated as *ns, not significant*.

Obesity and intestinal dysbiosis are not only interlinked, but also important drivers of chronic inflammation (32–38). In accordance with that and apart from a mere increase in WAT mass (**Figure 3B**) and leptin transcripts (**Figure 4A**), LF-treated animals displayed signs of exacerbated fat inflammation as shown by significantly increased inflammatory *IL-1β* and *TNFα*, and slightly increased *IL-6 mRNA* expression (**Figure 4A**). A subset of inflammatory markers was found to be elevated systemically as well. In LF-mice, we found increased leptin serum concentrations along with significantly higher levels of GM-CSF, TNFα, IL-6 and MCP1 while IFNγ, IL-23, IL-10 and IL-17 showed only a slight, not significant increase (**Figure 4B**).

To examine if typical pro-inflammatory cytokines might affect adaptive immune activation and link chronic inflammation to disease pathology, we treated 20w old NZB/WF1 animals over a period of 6 weeks with a mix of recombinant TNFα, IL-6 and IL-1β. Compared to PBS-treated control animals, continuous cytokine application significantly increased lymphoproliferation and CD44 expression on CD8, but not CD4 T cells. We also noted a more pronounced, although not significantly increased production of anti-dsDNA-IgG (**Figure 4C**). While these results largely support our assumptions, they do not allow a clear distinction between adaptive immune activation being directly triggered by inflammatory cytokines versus developing along with more quickly progressing disease.

Altogether, our results indicate that fiber intake impacts intestinal integrity, energy homeostasis and weight regulation. These may synergistically propagate a state of chronic, systemic, low-grade inflammation, tipping the balance towards an accelerated autoimmune pathology along with a pro-inflammatory immune-phenotype.

### Human Obesity Is Associated With Intestinal Leakage and Systemic Inflammation While Adaptive Immune Activation Shows a Predominant Correlation With SLE Activity

To strengthen the assumption of the linkage of obesity with intestinal leakage, systemic inflammation and adaptive immune activation as potential drivers of disease pathology, we further examined this connection in SLE patients.

**Figure 5:**
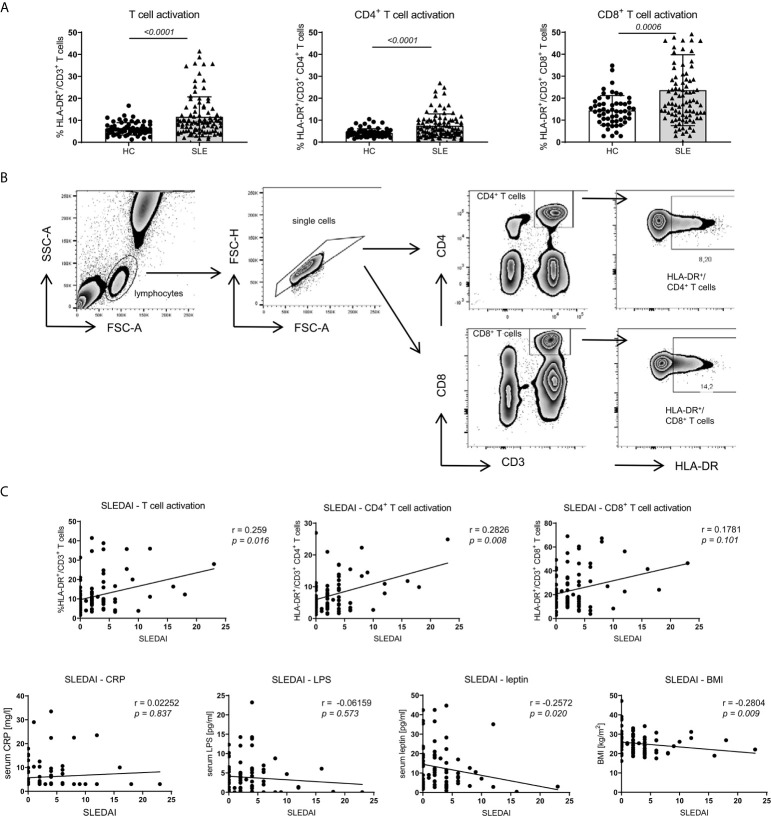
Association between SLE activity, obesity, intestinal leakage, inflammation and adaptive immune activation. **(A)** Comparison of HLA-DR expression as marker of immune activation on CD3^+^, CD3^+^CD4^+^ and CD3^+^CD8^+^ T cells in SLE patients (n = 86) and healthy controls (HC; n = 67). Results are expressed as scatter blots with mean ± SEM; each data point represents an individual donor; *p <0.05* was considered significant. **(B)** Representative FACS-blots indicating the selection of lymphocytes, doublet-exclusion, selection of CD3^+^CD4^+^ or CD3^+^CD8^+^ T cells and expression analysis of HLA-DR. **(C)** The relationship between disease activity (SLEDAI) and obesity (BMI and serum leptin), systemic inflammation (CRP), gut leakage (serum LPS) as well as adaptive immune activation (% HLA-DR expression on CD3^+^, CD3^+^CD4^+^ and CD3^+^CD8^+^ T cells) was analyzed by Spearman’s rank correlation; graphed are numerical values, Spearman’s rank correlation coefficient and its strength.

Compared to healthy controls (HC) and according to our observations in NZB/WF1 mice (**Supplementary Figure 2A**; **Supplementary Table 2**), SLE patients displayed significantly increased immune activation, determined by expression of HLA-DR on CD3^+^CD4^+^, CD3^+^CD8^+^ and whole CD3^+^ T cells (**Figures 5A, B**). In SLE patients, the level of immune activation of CD4^+^ and whole T cells clearly, of CD8^+^ T cells slightly correlated with disease activity, determined by SLEDAI (**Figure 5C** and **Table 1**).

**Table 1:** Association between SLE activity, obesity, intestinal leakage, systemic inflammation and adaptive immune activation.

		SLEDAI	BMI		
		Correlation^A^	Correlation^B^		
		SLE	HC	SLE	SLE sub
BMI	r	−0.2804			
	*p*	*0.009*			
leptin	r	−0.2572	0.7529	0.5876	0.7143
	*p*	*0.02*	*<0.001*	*<0.001*	*<0.001*
CRP	r	0.02252	0.5874	0.2257	0.3402
	*p*	*0.837*	*<0.001*	*0.038*	*0.071*
LPS	r	−0.06159	0.4437	0.105	0.386
	*p*	*0.573*	*<0.001*	*0.339*	*0.039*
%HLA-DR/CD3^+^ T cells	r	0.259	−0.07666	−0.05047	0.197
	*p*	*0.016*	*0.547*	*0.646*	*0.306*
%HLA-DR/CD3^+^CD4^+^ T cells	r	0.2826	−0.007213	−0.007213	0.05172
	*p*	*0.008*	*0.955*	*0.136*	*0.79*
%HLA-DR/CD3^+^CD8^+^ T cells	r	0.1781	−0.05827	−0.1629	0.3542
	*p*	*0.101*	*0.647*	*0.136*	*0.059*

^A^SLEDAI correlation: Association between disease activity (SLEDAI) and obesity (BMI, serum leptin), systemic inflammation (CRP), gut leakage (serum LPS) as well as adaptive immune activation (% HLA-DR expression on CD3^+^, CD3^+^CD4^+^ and CD3^+^CD8^+^ T cells) in all enrolled SLE patients (n = 86).

^B^BMI correlation: Association between obesity (BMI) and leptin, systemic inflammation (CRP), gut leakage (serum LPS) as well as adaptive immune activation (% HLA-DR expression on CD3^+^, CD3^+^CD4^+^ and CD3^+^CD8^+^T cells) in healthy controls (HC; n = 67), all enrolled SLE patients (SLE; n = 86) and a defined group of SLE patients with SLEDAI ≤2, receiving a treatment of no more than hydroxychloroquine/chloroquine, prednisone ≤5 mg and no DMARDs/biologicals (SLE sub; n = 29).

^A,B^Graphed are numerical values, Spearman’s rank correlation coefficient and its strength.

To explore a possible association of disease activity with systemic inflammation, obesity and intestinal leakage, we determined serum levels of the acute phase reactant C-reactive protein (CRP), serum lipopolysaccharide (LPS) as indirect measure of intestinal leakage as well as body mass index (BMI) and leptin serum levels as measure of obesity. In contrast to the positive association between SLEDAI and immune activation, we did not find a correlation between disease activity (SLEDAI) and CRP or LPS levels and even a negative correlation with BMI and leptin (**Figure 5C** and **Table 1**). These results might be explained by a) CRP in general not being a typical SLE activity marker; b) the confounding effects of different therapeutic regimes with usually highest corticosteroid doses on top of treatment with biologicals and DMARDs in patients with elevated disease activity, which is also found in our study (**Supplementary Table 4**); c) weight loss being a frequent clinical sign of active disease (B symptoms).

Therefore, we decided to examine these associations also in healthy controls (HC) and in a more defined group of lowly active SLE patients (‘SLE subgroup’) with a SLEDAI ≤2, receiving a treatment of not more than hydroxychloroquine/chloroquine and/or prednisone ≤5 mg and no immunosuppressants or biologicals. Generally, for all SLE patients, HC and the ‘SLE subgroup’ we observed a positive and mostly significant correlation of obesity (BMI) with leptin and CRP, in HC and the ‘SLE subgroup’ additionally between BMI and LPS levels (**Table 1**). Generally, the observed correlations were strongest for HC, possibly due to the fact, that there are no or less confounding effects from disease, co-morbidities or medication.

In contrast to the overall association between obesity, intestinal leakage and low-grade inflammation, we did not find a clear correlation between obesity and markers of immune activation (**Table 1**). Only within the subgroup of SLE patients, the BMI correlated positively with immune activation in CD8^+^ T cells, while there was no such correlation for CD4^+^ and whole CD3^+^ T cells. No correlation and rather a slightly negative association was found between BMI and immune activation in the group of all enrolled SLE patients and HC (**Table 1**). With regard to the overall weak or lacking correlation between BMI and immune activation, it needs to be considered, that: a) immune activation shows a clear association with SLE activity/SLEDAI; consequently, immune activation is generally low in HC and the lowly active ‘SLE subgroup’. b) The lack of correlation in all SLE patients is likely explained by the negative correlation between disease activity and BMI, with weight loss being a frequent clinical sign of active disease (B symptoms) (**Figures 5A, C** and **Table 1**).

Altogether, these results partly support our murine data. They strengthen the hypothesis of an association of obesity with intestinal leakage and systemic inflammation. That clear signs of elevated adaptive immune activation were predominantly found in SLE patients with active disease, points out the requirement of stimuli such as antigen exposure. Although a clear association between obesity and immune activation could not be established, it cannot be excluded, that in the setting of SLE, factors like obesity might additionally push adaptive immune activation and sustain disease activity. A further limitation is that in this human cohort, we were not able to explore the link between fiber intake and obesity, reported in mice.

### Neither Gut Microbiota Suppression, nor SCFA Feeding Exert Predominant Effects on Disease Pathogenesis in NZB/WF1 Mice

**Figure 6:**
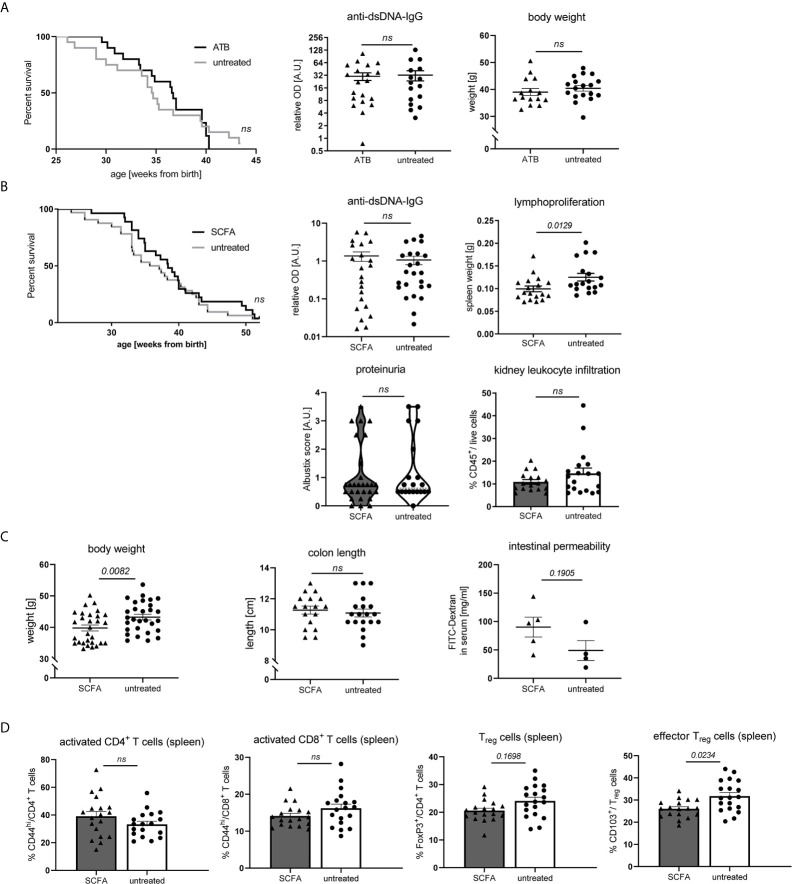
Impact of SCFA application or microbiota suppression on disease development in NZB/WF1 animals. **(A)** Lupus-prone NZB/WF1 mice were administered antibiotics (ATB) in the drinking water (n = 15–20 mice) versus untreated drinking water (n = 18–20 mice). Overall survival as well as anti-dsDNA autoantibodies and body weight were determined at 28 w. **(B–D)** Lupus-prone NZB/WF1 mice received a SCFA-mix or untreated drinking water. **(B)** Overall survival (n SCFA = 27 mice, n untreated = 32 mice), anti-dsDNA autoantibodies, proteinuria and kidney infiltration by leukocytes, as well as spleen weights as sign of lymphoproliferation were determined at 28 w (n SCFA = 17–22 mice, n untreated = 18–24 mice). **(C)** Body weight (n SCFA=26-29 mice, n untreated = 29 mice), colon length (n SCFA = 18 mice, n untreated = 19 mice) as well as intestinal permeability with FITC-Dextran applied by oral gavage, were determined at 28 w (n SCFA = 5 mice, n untreated = 4 mice; representative of two independent experiments). **(D)** Broad immune status evaluation in spleen of 28 w old NZB/WF1 mice receiving a SCFA-mix (n = 18) or untreated drinking water (n = 19). Depicted are CD44^hi^ expression on CD4^+^ and CD8^+^ as marker of T cell activation as well as frequencies of FoxP3^+^ T_reg_ and CD103^+^FoxP3^+^ effector T_reg_. The Kaplan–Meier method was used for estimating OS in differently treated groups. All other results are expressed as scatter blots with mean ± SEM; each data point represents an individual mouse; *p < 0.05* was considered significant, *p > 0.2* is indicated as *ns, not significant*.

As SCFA and bacterial metabolism are influenced by dietary fiber intake and can affect inflammatory diseases on various levels, we also set out to explore to what extent their actions might influence disease pathology in our model. To that end, NZB/WF1 mice were continuously fed a mix of the most abundant SCFA (Acetate, Butyrate and Propionate). To assess the role of gut microbiota, we suppressed bacterial growth by application of broad spectrum antibiotics. Antibiotics treatment affected neither OS, nor autoantibody production or weight regulation (**Figure 6A**). In addition, we did not find clearly beneficial effects when feeding SCFA. Overall, SCFA feeding hardly improved OS, while it decreased spleen weights. No differences were found for proteinuria as well as anti-dsDNA-IgG levels (**Figure 6B**). Apart from an increase in T_reg_ and effector T_reg_ frequencies, no immune-phenotypical changes were observed upon SCFA treatment (**Supplementary Table 5** and **Figure 6D**). Like in HF-fed mice, but less pronounced, SCFA-treated animals presented lower body weights, however there were no differences in intestinal leakage (**Figure 6C**).

Altogether, these results suggest that SCFA-feeding does not exert significantly beneficial effects on lupus pathology in our model. In support of that, also systemic SCFA application (data not shown) or maternal SCFA feeding during pregnancy and lactation (data not shown) had no effects.

## Discussion

The central question of this study was to elucidate whether a lack of fiber, as typically found in Western type diets, contributes to lupus pathogenesis and to unravel underlying mechanisms. We hypothesized that bacterial metabolism and especially the generation of SCFA might play a leading role by fortifying a healthy intestinal homeostasis and beneficial immunologic effects. This hypothesis was supported in some aspects by our results, as low intake of dietary fiber accelerated lupus pathology and the associated immune-dysregulation. Surprisingly, direct SCFA feeding did not influence lupus manifestation. Our data indicate that rather an increase in WAT mass and fat-inflammation link reduced fiber intake and lupus pathogenesis in our model. We hypothesize that this, combined with intestinal leakiness, inflicts a state of systemic low-grade inflammation accelerating disease development. These data not only add to the understanding how low fiber intake may propagate autoimmune pathogenesis, but also how intricately the pathologies of inflammatory immune mediated conditions, such as autoimmunity and obesity, are interlinked.

Generally, our study supports most published work in this area propagating an association between fiber intake and the pathogenesis of different immune-mediated inflammatory diseases such as allergic, autoimmune and cancerous conditions (20–25) and parameters of metabolic syndrome (39–41). In particular, our data support the connection between low fiber intake, obesity and intestinal leakage as drivers of inflammation and lupus pathology.

The here prevailing inverse association between fiber consumption and reduced body fat/obesity is supported by other studies (42). Generally, a myriad of different fiber effects might contribute, such as a changed microbiota (43–45), enhanced satiety and lower food intake, an accelerated gastro-intestinal transit along with increased energy absorption (42, 46–49), as well as changed SCFA contents influencing energy expenditure, appetite and weight *via* neural and humoral pathways (17–19). The lacking effect of antibiotics treatment argues against microbiota shifts playing a major role in weight development in our model. We hypothesize a major contribution by reduced energy exploitation in HF-fed mice as a consequence of an accelerated intestinal passage along with increased fecal bulking and laxation and enlarged intestinal lengths. The latter most probably results from the hygroscopic fiber effects increasing fecal volume and viscosity, posing a higher mechanical strain and stretch on the intestinal wall and stronger propulsive peristalsis (42, 47–49). Although not directly shown, increased food and energy intake by HF-fed animals further supports the postulated energy loss and argues against a connection between fiber intake and enhanced satiety as alternative anti-obesogenic mechanism (46, 50) in this study. Last but not least, SCFA feeding was associated with a slightly lower weight gain, possibly making an additional contribution to the lower weight gain in HF mice.

That obesity and a leaky gut, as found in our mice receiving a LF-diet, can propagate bacterial translocation and drive autoimmunity is widely accepted (35, 38). Apart from a translocation of specific autoimmunity-triggering pathobionts into systemic organs (13, 51), the generally invoked metabolic endotoxemia can stimulate multiple immune cells *via* TLRs and lead to systemic low-grade inflammation (32). Such a scenario has recently been suggested in lupus-prone FcGRIIb^−/−^ mice treated with gut leakage-inducing dextran sodium sulfate (DSS) enhancing systemic inflammation, apoptosis and lupus progression (52). In addition and like endotoxemia, WAT itself is an important inducer of local and systemic low-grade inflammation as confirmed in our study. WAT not only serves as energy depot, but can also release a plethora of pro-inflammatory cytokines, such as TNFα, IL-1β or IL-6 (53–55). In addition, adipocytes release ‘adipokines’ such as leptin, found to be elevated in our more obese LF-fed mice. Adipokines regulate effects such as satiety, energy expenditure and adipocyte metabolism (56, 57). Many of them also display pro-inflammatory properties and direct immune-regulatory effects. This not only contributes to the development of comorbidities (such as diabetes or cardiovascular disease), but could also worsen autoimmune pathogenesis (35, 58–61).

Diet shapes microbial communities in the gut influencing metabolic and immune responses (43–45). A fiber-mediated change in gut bacteria or SCFA composition was not specifically addressed here, but has been reported by a large number of previous publications (6, 23, 25, 62–66). Of course, this does not automatically imply that equivalent SCFA level changes and microbiota shifts prevail under the here employed conditions and using the NZB/W F1 strain. As colonic transit time not only regulates nutrient availability but also luminal wash out thereby shaping the microbiota ecosystem (67), microbiota-shifts appear probable also in our study and might propagate weight gain and/or affect the intestinal barrier function (6, 7, 13, 33). In contrast to that, fiber-associated changes of the intestinal barrier function are described to occur independently from cecal bacterial profiles (29) and could, for instance, be inflicted by a simple resorting of the gut microbiota to host-secreted mucus glycoproteins serving as a nutrient source during fiber deficiency (68). We found that low fiber intake reduced intestinal integrity along with the expression of molecules associated with mucus layer and epithelial repair. The lack of stringent effects of antibiotic treatment on lupus pathology in our study might argue against major microbiota-dependent effects in the given setting, it does however not exclude an involvement of the microbiome in disease-accelerating effects of the LF-diet. A limitation of this study is that this was not further addressed here. Feeding different diets with or without antibiotics and fecal transfer experiments might be required to solve this question. Additionally, such follow-up studies should consider and examine the impact of fiber-associated obesity as influencing variable on microbiota composition.

Likewise, feeding a SCFA mix did not significantly affect lupus pathology. In consideration of the frequently reported association between nutritional fiber contents and SCFA levels in feces and serum (6, 25), this is in accordance with our observation that a HF- compared to NF-diet only minimally ameliorated lupus progression. Thus, our results contrast with other studies (13, 69–71). While the cause underlying these differing outcomes of SCFA feeding and microbiota depletion on lupus pathology remains unclear, different factors might contribute. These include discrepancies in feeding regimes of specific fibers and SCFA, physicochemical characteristics of different fibers such as fermentability, solubility, and viscosity, animal facilities, as well as mouse strains and individual or strain-specific effects of the gut microbiota (13, 51, 66, 71–77). In support of the latter and mimicking the diversity in SLE patients, no simple overarching principle regarding the gut—microbiota—lupus connection has been found in lupus-prone strains so far (13, 78, 79).

Apart from strain- or feeding-specific circumstances and limitations, it needs to be considered that depending on the disease context, SCFA may even deteriorate disease pathology. It is becoming increasingly clear that the impact of SCFA on immune cells is not always merely anti-inflammatory (16). In CIA arthritis and experimental autoimmune encephalitis (EAE), early SCFA application could reduce disease severity, while increasing K/BxN serum transfer arthritis (22). Apart from T_reg_, long-term SCFA application was shown to trigger urethritis-inducing T_H_1 and T_H_17 cells when applied at higher than physiological levels (80). Such clearly pro-inflammatory effects were not noted in our studies, however it cannot be excluded they backfire anti-inflammatory actions leading to only minimal effects on the overall disease course. Furthermore, reported immunologic SCFA effects are not always consistent between studies, i.e. on T_reg_ frequencies and differentiation or on T_FH_, plasma cell differentiation and Ig production (7, 14, 15, 22–25, 69, 80–82). As SCFA effects may contextually vary, their influence in relation to different micro-environmental conditions and multicomponent disease states needs further exploration.

To sum up, in accordance with others, our murine study shows a clear association between reduced fiber intake and autoimmune pathology and a connection with systemically elevated cytokine levels, obesity, fat inflammation, gut leakage and immune-dysregulation (6, 7, 12, 13, 32–38, 83). The association between obesity and autoimmunity is generally recognized for different entities and may suggest a more general concept as well as synergism effects in the pathology of inflammatory, immune-mediated diseases.

In the human cohort, we could not explore the link between fiber intake and obesity reported in mice, but confirm an association between obesity, intestinal leakage and systemic inflammation, while adaptive immune activation was mainly associated with SLE disease activity. The lack of a clear correlation between obesity and immune activation does however not exclude, that in a setting of persistent antigen exposure, such as in chronic infections, autoimmune disease or cancer, systemically increased inflammatory cytokines, e.g. as a consequence of bacterial translocation or obesity, may additionally drive lymphocyte activation and disease progression. Such a scenario has been reported for HIV and also revealed a correlation with markers of cardiovascular comorbidity (84, 85). An increased risk of cardiovascular mortality related to a dysregulated innate and adaptive immune response was also reported in SLE (86).

Although not directly addressed here, we assume that the suspected multifactorial and synergistically acting factors may be bidirectionally linked, which is supported by previous reports. For instance, obesity is strongly associated with changes in the gut microbiome, gut leakage and bacterial translocation. Vice versa, WAT contributes to elevated levels of pro-inflammatory cytokines and adipokines, that—among other things—can induce apoptosis of enterocytes, disrupt tight junctions and contribute to gut dysbiosis and bacterial translocation (11, 33, 34, 36, 37, 63, 87, 88). The sharing of such common pathways is reflected in the clustering of comorbidities such as obesity, metabolic syndrome, diabetes or cardiovascular complications and the close connection with SLE/autoimmunity (35, 38, 86, 89, 90). Therefore, it is important to understand their molecular links and how these are influenced by dietary factors. Given the high rise in inflammatory and autoimmune diseases and associated mortality, the translation of such findings in effective therapies and prevention strategies, might not only improve individual suffering, but also have an important socio-economic impact.

## Data Availability Statement

The original contributions presented in the study are included in the article/**Supplementary Material**. Further inquiries can be directed to the corresponding author.

## Ethics Statement

The studies involving human participants were reviewed and approved by Ethikkommission Universität Freiburg. The patients/participants provided their written informed consent to participate in this study. The animal study was reviewed and approved by Regierungspräsidium Freiburg.

## Author Contributions

NC designed research. NC, A-LS, AE, DS, AA, CD, CF, and ANK performed research. NC, A-LS, AE, CH, CD, SF, MR, ANK, CF, and RV analyzed data. US contributed new reagents/analytical tools. NC, A-LS, CD, MR, and RV contributed to manuscript editing. NC and A-LS wrote the paper. All authors contributed to the article and approved the submitted version.

## Funding

This work was supported by the B. Braun-Foundation (Germany), Müller-Fahnenberg-Stiftung (Albert-Ludwig-University of Freiburg, Germany), the Research Committee (Forschungskommission) of the Medical Faculty of the University of Freiburg, the Ministry of Science, Research, and Arts Baden-Wurttemberg (Margarete von Wrangell Programm to NC), Deutsche Forschungsgemeinschaft (DFG) (TRR 130, project 12 to RV) and Dr. Heinrich Kircher Foundation (Albert-Ludwigs-University of Freiburg, Germany; to AE and AA).

## Conflict of Interest

The authors declare that the research was conducted in the absence of any commercial or financial relationships that could be construed as a potential conflict of interest.
