# Supplementary material for: Low Dietary Fiber Intake Links Development of Obesity and Lupus Pathogenesis
Source: Front Immunol. 2021 Jul 15;12:696810. doi: 10.3389/fimmu.2021.696810 (PMC8320762; doi:10.3389/fimmu.2021.696810)
Supplement: Supplementary file 1 [file DataSheet_1.pdf]

Supplementary Material

Supplementary Methods

Adoptive transfer experiments

Cell suspensions were prepared from pooled spleens and lymph nodes of NZB/WF1 mice without nephritis. T cells were enriched by negative selection using MACS microbeads (Miltenyi Biotech) and stained with CFDA-SE (CFSE) (Thermofisher). 2.5x 10^6 labelled cells were adoptively transferred into healthy (14 week old) or nephritic NZB/WF1 animals. 24 hours later, recipient mice were sacrificed for collection of kidneys to determine the frequencies of transferred $CFSE^+$ $CD4^+$ and $CD8^+$ T cells by flow cytometry.

Supplementary Figures

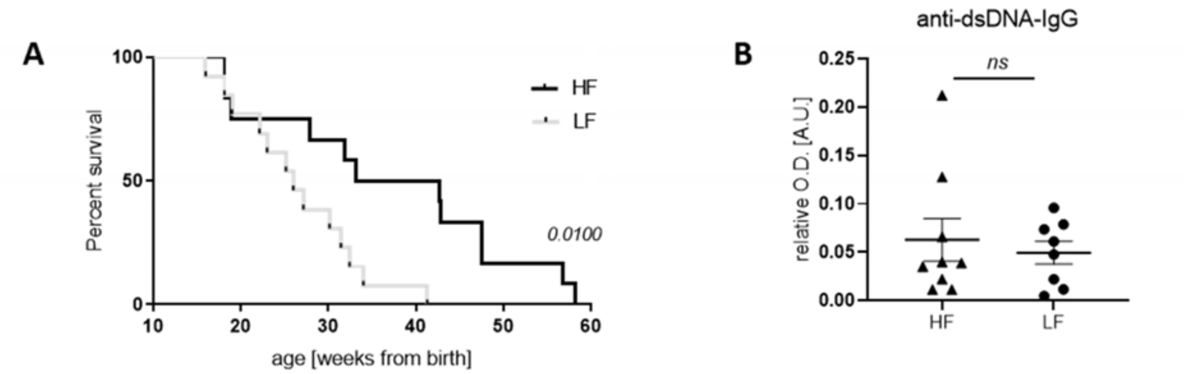

Supplementary Figure 1. Impact of dietary fiber on lupus pathology in MRL/*lpr* mice

(A) Overall survival (OS) determined in lupus-prone MRL/*lpr* mice fed a high (HF; n=12) or low fiber (LF; n=13) diet. The Kaplan-Meier method was used for estimating OS in differently treated groups. **(B)** Measurement of anti-dsDNA-IgG serum titers in 20w old animals fed a HF (n=9 mice) or LF (n=8 mice) diet. Results are expressed as scatter blots with mean +/-SEM; each data point represents an individual mouse; *p<0.05* was considered significant, *p>0.2* is indicated as *ns, not significant*.

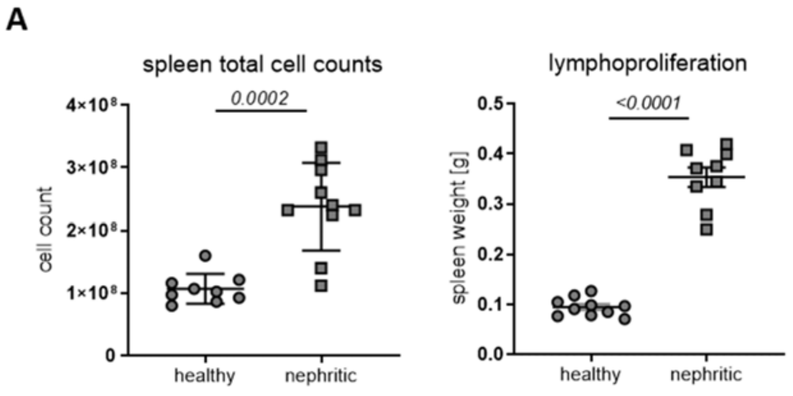

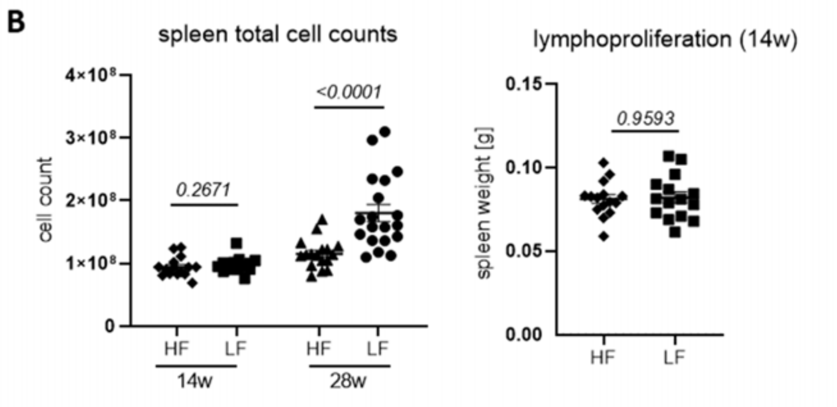

Supplementary Figure 2. Lymphoproliferation in progressing disease

(A) Spleen weights and total splenocyte counts in 14w old NZB/WF1 mice (healthy, n=9-10 mice) versus mice with established nephritis (nephritic, n=9-10 mice) fed a normal chow. **(B)** Total splenocyte counts in 14w (n HF=15 mice, n LF=15 mice) versus 28w (n HF=17 mice, n LF=19 mice) old NZB/WF1 mice fed a HF- versus LF-diet; spleen weights of 14w old NZB/WF1 mice fed a HF- versus LF-diet (n HF=15 mice, n LF=15 mice). Results are expressed as scatter blots with mean +/- SEM; each data point represents an individual mouse; *p<0.05* was considered significant, *p>0.2* is indicated as *ns, not significant*.

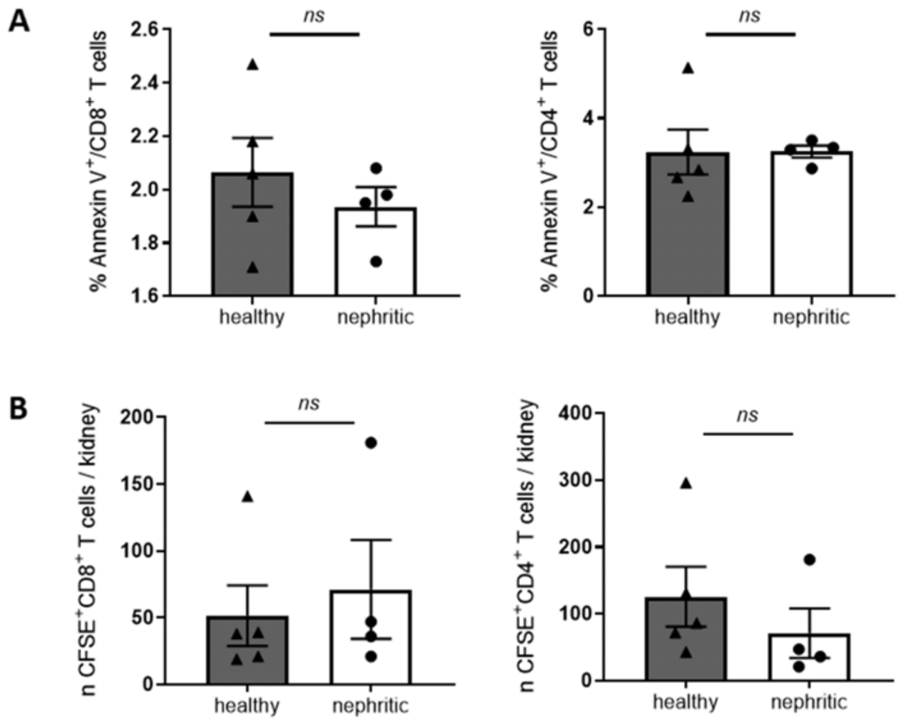

Supplementary Figure 3. Susceptibility to apoptosis and migration of CD4^{+} and CD8^{+} T cells into inflamed and non-inflamed kidneys

(A) Annexin V-binding was examined in spleen CD4^{+} and CD8^{+} T cells from healthy (14w old, n=5 mice) versus nephritic NZB/WF1 animals (n=4 mice). **(B)** Number of CFSE-labelled CD4^{+} and CD8^{+} T cells, recovered from one kidney of healthy (14w old, n=5 mice) versus nephritic NZB/WF1 animals (n=4 mice) 24h after adoptive transfer. Results are expressed as scatter blots with mean +/-SEM; each data point represents an individual mouse; *p<0.05* was considered significant, *p>0.2* is indicated as *ns, not significant*.

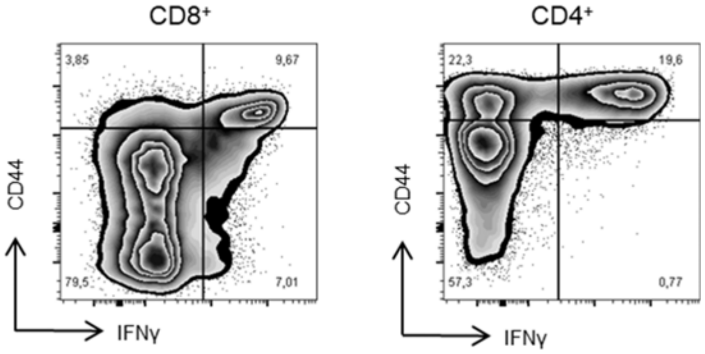

Supplementary Figure 4. Co-expression of CD44 and IFNγ in CD4 and CD8 T cells

Co-expression of CD44 and IFNγ in $CD4^+$ and $CD8^+$ T cells of 28w old NZB/WF1 mice by flow cytometry. Shown are representative FACS blots from spleen.

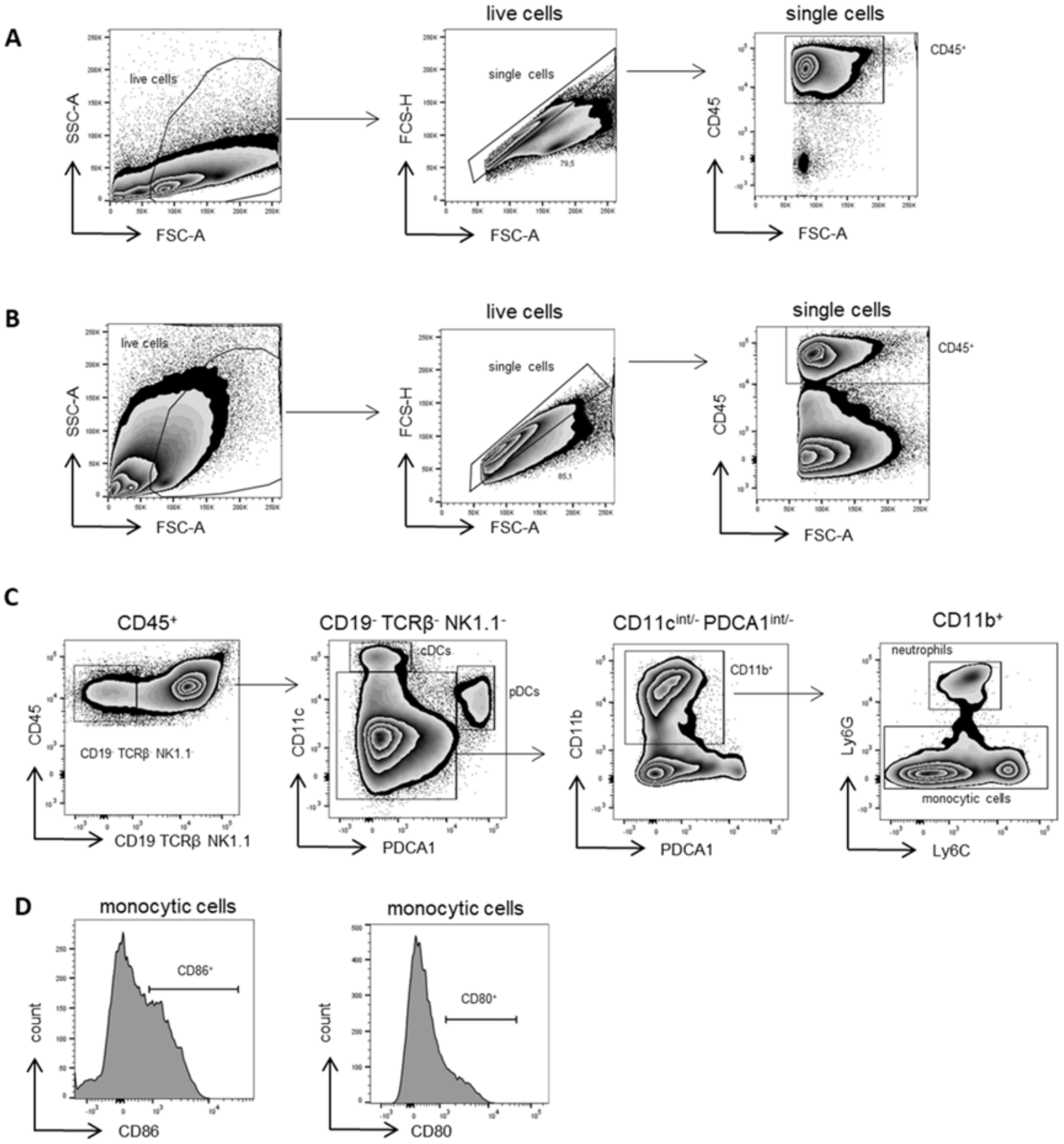

Supplementary Figure 5. Gating strategy for immune status evaluation (innate immune cells)

(A, B) After FSC/SSC-based selection of live leukocytes and exclusion of doublets, CD45^{+} organ-infiltrating neutrophils were selected. Shown are representative FACS blots from spleen (A) and kidney (B). **(C)** To determine frequencies of main innate immune cell populations, CD45^{+}CD19^{-}TCRβ^{-}NK1.1^{-} were selected and among those cDCs identified as CD11c^{hi}, pDCs as PDCA1^{hi} cells. Monocytic cells were identified as CD11c$^{int/-}$PDCA1$^{int/-}$CD11b^{+}Ly6G^{-}, neutrophils as CD11c$^{int/-}$PDCA1$^{int/-}$CD11b^{+}Ly6G^{hi} cells. Shown are representative FACS blots from spleen. **(D)** The expression of the co-stimulatory molecules CD86 or CD80 was quantified on monocytic cells (shown) or cDC (not shown).

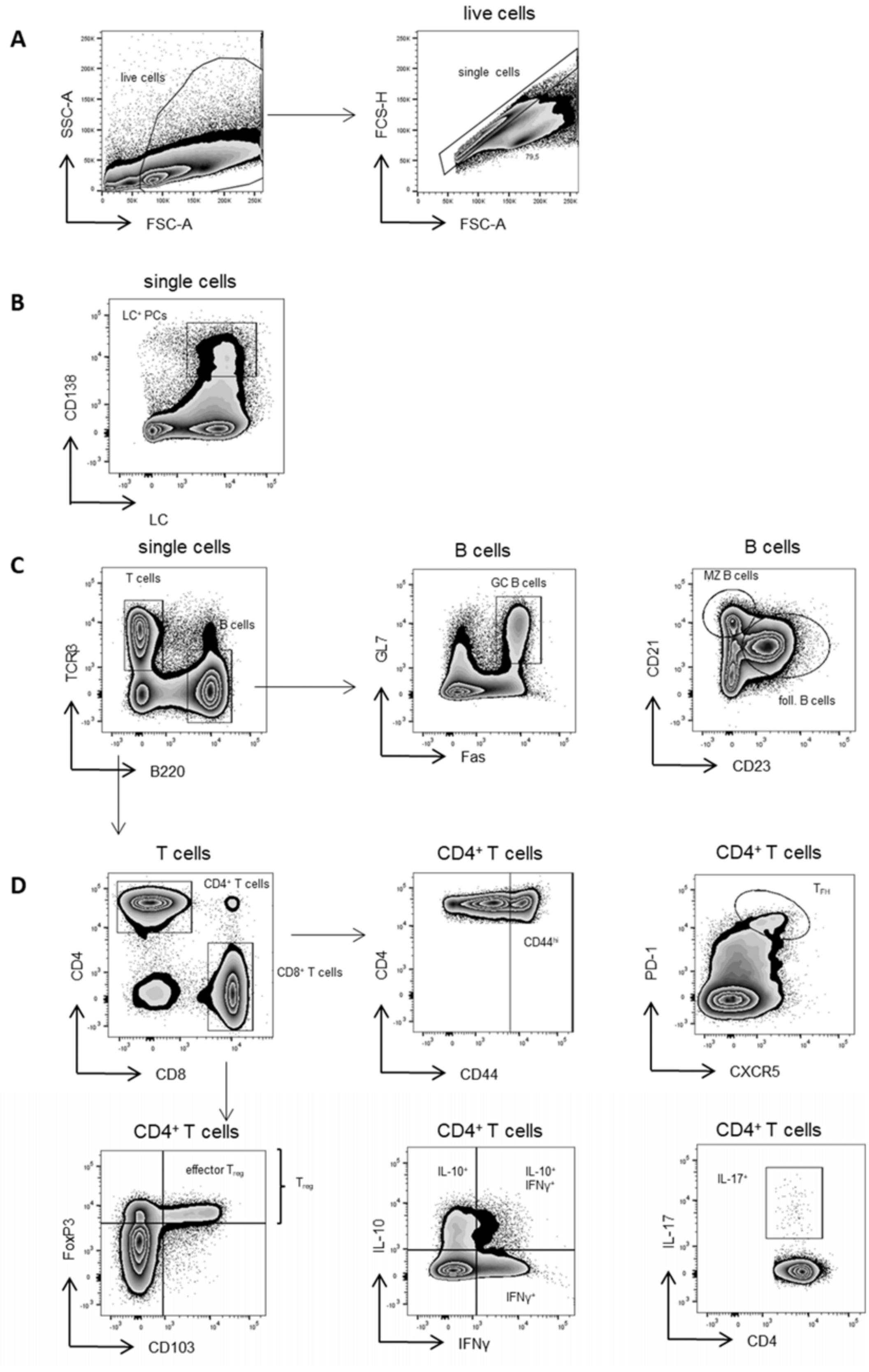
A
live cells
SSC-A
FSC-A
live cells
single cells
FCS-H
FSC-A
79,5
B
single cells
LC+ PCs
CD138
LC
C
single cells
T cells
B cells
TCRβ
B220
B cells
GC B cells
GL7
Fas
B cells
MZ B cells
foll. B cells
CD21
CD23
D
T cells
CD4+ T cells
CD8+ T cells
CD4
CD8
CD4+ T cells
CD44hi
CD4
CD44
CD4+ T cells
TFH
PD-1
CXCR5
CD4+ T cells
effector Treg
Treg
FoxP3
CD103
CD4+ T cells
IL-10+
IL-10+ IFNγ+
IFNγ+
IL-10
IFNγ
CD4+ T cells
IL-17+
IL-17
CD4

Supplementary Figure 6. Gating strategy for immune status evaluation (adaptive immune cells)

(A) After FSC/SSC-based selection of live leukocytes, doublets were excluded. **(B)** Plasma cells were identified as $CD138^{hi}\kappa/\lambda LC^{+}$ cells from single cells. **(C, D)** From single cells, B cells were selected as $TCR\beta^{-}B220^{+}$ cells (C), $CD4^{+}$ T cells as $B220^{-}TCR\beta^{+}CD8^{-}CD4^{+}$ (C, D) and $CD8^{+}$ T cells as $B220^{-}TCR\beta^{+}CD4^{-}CD8^{+}$ T cells (C, D). Further determined was the percentage of $GL7^{hi}Fas^{hi}$ GC B cells, $CD21^{hi}CD23^{lo}$ MZ B cells and $CD21^{lo}CD23^{hi}$ follicular B cells on whole B cells **(C)**. Determined was also the % expression of $CD44^{hi}$, IL-10, IFNg, IL-17, $CXCR5^{hi}PD1^{hi}$ T_{FH}, $FoxP3^{+}$ T_{reg} and $CD103^{+}/FoxP3^{+}$ effector T_{reg} on whole $CD4^{+}$ T cells (D). Shown are representative FACS blots from spleen.

Supplementary Tables

Supplementary Table 1. Immune status in HF- versus LF-fed NZB/WF1 animals

	14w			28w			sick		
	**HF**	**LF**		**HF**	**LF**		**HF**	**LF**	
**spleen**	mean +/- SEM	mean +/- SEM	*p-value*	mean +/- SEM	mean +/- SEM	*p-value*	mean +/- SEM	mean +/- SEM	*p-value*
CD11b$^+$Ly6G^{hi} neutrophils/live cells	0.1253	0.1113	*ns*	0.351	0.303	*ns*	0.3571	0.4771	*ns*
	0.03651	0.0155		0.08532	0.07805		0.09329	0.1194	
CD11c^{hi} cDC/ live cells	0.08	0.104	*0.0927*	0.1183	0.1882	*ns*	0.2105	0.5529	*0.0012*
	0.004629	0.01166		0.0339	0.01267		0.01495	0.1055	
CD80$^+$/cDC	84.92	85.53	*ns*	76.72	70.68	*0.0555*	62.14	49.6	*0.1014*
	1.445	1.413		4.262	2.397		6.456	5.382	
CD86$^+$/cDC	10.02	10.16	*ns*	7.268	9.903	*0.0027*	20.81	16.81	*0.1282*
	2.911	2.544		0.5572	0.5193		1.166	2.078	
PDCA1^{hi} pDC/live cells	0.161	0.228	*0.1474*	0.2203	0.1419	*0.011*	0.06571	0.09143	*0.2145*
	0.02822	0.03878		0.02802	0.02065		0.01798	0.01738	
Ly6G$^-$CD11b$^+$ monocytic cells/ live cells	2.557	2.215	*ns*	1.332	1.144	*0.0275*	1.076	1.373	*ns*
	0.3946	0.4646		0.06755	0.04852		0.2253	0.2865	
CD80$^+$/monocytic cells	44.09	38.82	*ns*	45.71	39.46	*0.0065*	57.3	47.46	*ns*
	4.473	4.055		1.94	0.9389		6.623	7.409	
CD86$^+$/monocytic cells	4.343	4.295	*ns*	10.88	11.43	*0.1312*	10.77	14.72	*0.0973*
	1.2	1.003		0.8417	0.5225		1.642	1.546	
TCRβ$^+$CD4$^+$ T cells/live cells	24.06	23.87	*ns*	24.84	19.86	*0.0035*	20.96	21.28	*ns*
	1.309	1,035		1.156	0.9716		1.392	1.85	
CD44^{hi}/CD4$^+$ T cells	12.2	12.88	*ns*	36.74	59.19	*<0.0001*	56.67	72.4	*0.0175*
	0.7192	1.539		3.092	3.877		7.282	2.204	
CXCR5^{hi}PD1^{hi}/ CD4$^+$ T cells (T_{FH})	0.48	0.3575	*ns*	2.884	5.516	*0.0015*	4.689	9.744	*0.0565*
	0.07974	0.03245		0.3821	0.5991		1.265	2.265	
IFNγ$^+$/CD4$^+$ T cells	3.164	3.462	*ns*	12.12	16.66	*0.02*	25.24	29.88	*ns*
	0.79	0.7842		1.38	1.333		3.477	3.421	
IL-17$^+$/CD4$^+$ T cells	0.232	0.11	*0.0556*	0.4123	0.4	*0.6239*	0.26	0.1957	*ns*
	0.04283	0.03017		0.06362	0.07092		0.05	0.03644	
IL-10$^+$/CD4$^+$ T cells	0.25	0.212	*ns*	3.373	6.759	*0.0003*	8.999	13.78	*0.0379*
	0.07993	0.05551		0.4721	0.7053		1.429	1.438	
FoxP3$^+$ /CD4$^+$ T cells (T_{reg})	14.38	13.24	*ns*	20.75	28.15	*0.0007*	22.3	27.64	*0.0787*
	1.766	0.595		1.267	1.272		2.121	1.233	
CD103$^+$/FoxP3$^+$CD4$^+$ T cells	13.63	14.32	*ns*	25.15	34.92	*0.0024*	38.94	53.05	*0.0111*
(effector T_{reg})	0.588	1.196		1.667	2.261		3.945	0.74	
TCRβ$^+$CD8$^+$ T cells/live cells	18.29	16.65	*ns*	14.47	9.967	*0.0028*	5.325	2.203	*0.0012*
	1.635	1.601		0.9333	9.967		0.5426	0.3542	
CD44^{hi}/CD8$^+$ T cells	4.622	3.594	*0.1903*	13.66	22.6	*0.0024*	10.42	21.83	*0.0070*
	0.5405	0.3901		0.9019	2.334		1.638	2.107	
IFNγ$^+$/CD8$^+$ T cells	12.9	12.47	*ns*	13.94	12.2	*0.3081*	27.1	47.36	*0.0221*
	1.776	2.523		1.038	0.9123		5.769	4.412	
TCRβ$^-$B220$^+$ B cells/live cells	44.11	56.83	*0.0952*	43.47	44.87	*ns*	46.57	40.04	*ns*
	2.898	9.722		1.735	1.678		2.872	5.356	
Fas^{hi}GL7^{hi}/B cells	0.4888	0.5225	*ns*	1.423	2.33	*ns*	4.545	4.694	*0.6163*
	0.09563	0.09955		0.2986	0.3123		0.4609	0.408	
CD21^{lo}CD23^{hi} (follicular)/ B cells	61.57	54.18	*ns*	61.73	57.04	*0.0118*	48.43	52.62	*ns*
	3.494	4.653		0.7922	1.387		5.979	1.744	
CD21^{hi}CD23^{lo} (MZ)/B cells	13.48	13.97	*0.8205*	11.55	11.02	*ns*	4.334	0.7443	*ns*
	1.379	0.8889		1.352	1.24		1.609	0.2154	
LC$^+$ CD138^{hi}/live cells (plasma cells)	1.062	0.742	*0.0601*	2.184	2.469	*ns*	2.091	2.023	*ns*
	0.1252	0.1155		0.2187	0.1813		0.3969	0.2869	
**kidney**									
CD45$^+$/live cells	10.26	14	*0.0393*	20.8	29.48	*0.1084*	68.72	76.49	*ns*
	1.127	1.396		4.249	4.42		5.191	1.447	
CD11b$^+$Ly6G^{hi} neutrophils/CD45$^+$ cells	3.722	2.735	*ns*	1.775	1.015	*0.0377*	1.204	1.103	*ns*
	1.133	0.6107		0.293	0.1645		0.3188	0.3165	
CD11c^{hi} cDC/CD45$^+$ cells	1.36	1.585	*ns*	0.3234	0.3564	*ns*	1.513	1.249	*ns*
	0.09663	0.1143		0.03083	0.04198		0.4775	0.2478	
PDCA1^{hi} pDC/CD45$^+$ cells	0.8036	0.9069	*ns*	0.4135	0.3356	*0.0663*	0.07333	0.1371	*0.1270*
	0.102	0.08902		0.0401	0.05396		0.03029	0.02523	
CD11b$^+$ monocytic cells/CD45$^+$ cells	19.95	24.87	*ns*	15.42	10.41	*0.0086*	12.27	13.71	*ns*
	3.03	3.36		1.383	0.8231		1.103	2.44	
TCRβ$^+$CD4$^+$ T cells/CD45$^+$ cells	19.72	22.79	*0.1656*	30.87	35.78	*0.0486*	33.05	35.77	*ns*
	1.642	0.9714		1.961	1.347		2.36	1.341	
CD44^{hi}/CD4$^+$ T cells	14.2	12.78	*0.1447*	39.73	47.88	*0.3147*	21.68	20.37	*ns*
	1.31	1.29		3.209	4.619		1.448	1.009	
IFNγ$^+$/CD4$^+$ T cells	3.921	5.964	*0.0439*	23.85	13.42	*0.0439*	17.91	13.58	*ns*
	0.6832	0.8176		3.66	1.758		2.753	2.112	
FoxP3$^+$/CD4$^+$ T cells (T_{reg})	14.85	13.71	*ns*	15.13	17.62	*0.037*	19.06	19.29	*ns*

	1.112	1.169		0.5649	0.8359		0.32	1.319	
CD103$^+$FoxP3$^+$/CD4$^+$ T cells	22.19	24.92	*ns*	22.57	24.67	*0.2711*	25.44	39.22	*0.0175*
(effector T_{reg})	1.882	1.891		1.353	1.357		2.889	3.297	
TCRβ$^+$CD8$^+$ T cells/CD45$^+$ cells	9.799	8.852	*0.1086*	11.48	18.12	< *0.0001*	23.11	23.83	*ns*
	0.848	0.9873		0.7375	0.9958		2.951	3.701	
CD44^{hi}/CD8$^+$ T cells	3.958	3.197	*0.1112*	16.68	25.19	*0.3342*	17.33	11.39	*0.1649*
	0.4138	0.2133		2.914	4.392		2.619	2.708	
IFNγ$^+$/CD8$^+$ T cells	7.755	12.36	*0.0688*	16.05	14.59	*ns*	26.66	31.15	*ns*
	1.405	2.025		1.3071	1.391		4.321	8.403	
TCRβ$^-$B220$^+$ B cells/CD45$^+$ cells	13.53	11.45	*ns*	11.53	10.76	*ns*	8.344	9.524	*ns*
	1.938	1.27		0.7848	1.169		1.205	0.82	

Broad immune status evaluation in spleen and kidney of HF- and LF-treated NZB/WF1 mice by FACS at 14w (yet healthy animals; n HF/LF=10-15 mice), at 28w (animals with established autoantibodies but no signs of overt nephritis; n HF=11-17 mice, n LF=14-19 mice) and in nephritic animals (n HF/LF=7 mice). Results are expressed as mean +/- SEM; $p<0.05$ was considered significant, $p>0.2$ is indicated as *ns, not significant*.

Supplementary Table 2. Immune status in healthy and nephritic NZB/WF1 animals

	healthy	nephritic	
**spleen**	mean +/- SEM	mean +/- SEM	*p-value*
$CD11b^+Ly6G^{hi}$ neutrophils/live cells	0.1586<br>0.06162	0.4425<br>0.09821	*0.0275*
$CD11c^{hi}$ cDC/ live cells	0.09<br>0.01215	0.325<br>0.04372	*<0.0001*
$CD80^+$/cDC	85.5<br>1.182	55.66<br>3.795	*<0.0001*
$CD86^+$/cDC	15.78<br>2.449	20.64<br>1.655	*0.1422*
$PDCA1^{hi}$ pDC/live cells	0.18<br>0.01215	0.07818<br>0.0122	*0.0005*
$Ly6G^-CD11b^+$ monocytic cells/ live cells	1.657<br>0.4879	1.431<br>0.2542	*ns*
$CD80^+$/monocytic cells	47.33<br>4.1	54.43<br>3.169	*ns*
$CD86^+$/monocytic cells	6.986<br>0.4731	11.47<br>1.017	*0.0019*
$TCR\beta^+CD4^+$ T cells/live cells	23.37<br>1.446	19.33<br>1.339	*0.0683*
$CD44^{hi}/CD4^+$ T cells	9.294<br>0.4639	71.1<br>2.299	*<0.0001*
$CXCR5^{hi}PD1^{hi}/ CD4^+$ T cells (T_{FH})	0.3743<br>0.01251	6.92<br>0.9928	*<0.0001*
$IFN\gamma^+/CD4^+$ T cells	3.489<br>0.4959	24.55<br>2.963	*<0.0001*
$IL\text{-}17^+/CD4^+$ T cells	0.3057<br>0.07606	0.1508<br>0.02013	*0.1146*
$IL\text{-}10^+/CD4^+$ T cells	0.3557<br>0.05851	11.77<br>1.191	*<0.0001*
$FoxP3^+ /CD4^+$ T cells (T_{reg})	14.63<br>1.115	25.49<br>0.7015	*<0.0001*
$CD103^+/FoxP3^+CD4^+$ T cells (effector T_{reg})	12.1<br>1.391	49.92<br>2.208	*<0.0001*
$TCR\beta^+CD8^+$ T cells/live cells	19.65<br>0.7801	3.564<br>0.4562	*<0.0001*
$CD44^{hi}/CD8^+$ T cells	3.19<br>0.2959	13.67<br>1.388	*0.0001*
$IFN\gamma^+/CD8^+$ T cells	9.184<br>0.5472	42.35<br>3.74	*<0.0001*
$TCR\beta^-B220^+$ B cells/live cells	45.85<br>1.136	43.07<br>3.741	*ns*
$Fas^{hi}GL7^{hi}$/B cells	0.4771<br>0.1386	2.248<br>0.3478	*0.0002*
$CD21^{lo}CD23^{hi}$ (follicular)/ B cells	62.11<br>3.057	43<br>4.795	*0.0221*
$CD21^{hi}CD23^{lo}$ (MZ)/B cells	9.281<br>1.545	2.643<br>0.6124	*0.0007*
LC^+ $CD138^{hi}$/live cells (plasma cells)	0.6714<br>0.06382	2.451<br>0.454	*0.0098*
**kidney**			
$CD45^+$/live cells	26<br>3.411	75.34<br>1.955	*<0.0001*
$CD11b^+Ly6G^{hi}$ neutrophils/$CD45^+$ cells	4.503<br>1.291	0.7033<br>0.09037	*0.1361*
$CD11c^{hi}$ cDC/$CD45^+$ cells	1.483<br>0.1407	1.14<br>0.1795	*0.1609*
$PDCA1^{hi}$ pDC/$CD45^+$ cells	0.7814<br>0.1154	0.1383<br>0.0286	*<0.0001*
$CD11b^+$ monocytic cells/$CD45^+$ cells	23.32<br>4.776	13.56<br>0.9018	*0.1422*
$TCR\beta^+CD4^+$ T cells/$CD45^+$ cells	25.13<br>1.988	31.34<br>1.399	*0.0358*
$CD44^{hi}/CD4^+$ T cells	7.344<br>0.4249	20.4<br>1.02	*<0.0001*
$IFN\gamma^+/CD4^+$ T cells	4.317<br>0.6084	17.1<br>3.329	*0.0221*
$FoxP3^+/CD4^+$ T cells (T_{reg})	14.14<br>1.128	19.47<br>1	*0.0052*
$CD103^+FoxP3^+/CD4^+$ T cells (effector T_{reg})	17.42<br>1.009	29.5<br>2.95	*0.0037*
$TCR\beta^+CD8^+$ T cells/$CD45^+$ cells	8.73<br>0.6389	30.14<br>1.84	*<0.0001*

CD44^{hi}/CD8^{+} T cells	3.643	14.76	*0.0018*
	0.911	2.88	
IFNγ^{+}/CD8^{+} T cells	12.95	28.13	*0.0831*
	4.612	5.358	
TCRβ^{-}B220^{+} B cells/CD45^{+} cells	12.32	8.646	*0.0866*
	1.776	1.173	

Broad immune status evaluation in spleen and kidney of 14w old, yet healthy and nephritic animals fed a normal chow (n healthy=7-9 mice, n nephritic=10-12 mice). Results are expressed as mean +/- SEM; *p<0.05* was considered significant, *p>0.2* is indicated as *ns, not significant*.

Supplementary Table 3. Patient's and healthy control's characteristics

	HC	SLE	SLE subgroup
**total number**	**67**	**86**	**29**
females	60	71	27
males	7	15	2
**age (years)**			
median	46	48	52
range	18-64	19 - 83	19-82
**BMI (kg/m^2)**			
median	29,3	24.5	25,6
range	18,5-65	16,1 - 47,2	18,6-47,2
**Leptin (pg/ml)**			
median	18.75	9,07	12,3
range	2,09-55,28	0,36-44,67	1,8-42,5
**CRP (mg/l)**			
median	3	3	3
range	3-28,8	3-33,5	3,0-29
**LPS (pg/ml)**			
median	2.56	2.58	1.3
range	0-19.78	0-23.22	0-12.05
**SLEDAI**			
median	/	2	1
range	/	0 - 23	0-2
**%HLA-DR/CD3$^+$ T cells**			
median	5,7	8,96	9
range	1,25-12,2	1,63-41,42	4-34,7
**%HLA-DR/CD4$^+$ T cells**			
median	3,68	6	5,9
range	1,52-9,22	1,27-26,93	21-26,9
**%HLA-DR/CD8$^+$ T cells**			
median	13,96	18,51	21,3
range	2,71-34,8	2,9-69,01	2,9-54,9

Depicted are total numbers of subjects divided by male and female, median (+ range) age (years), BMI (kg/m^2), serum levels of leptin (pg/ml), CRP (mg/l), LPS (pg/ml), SLEDAI and % HLA-DR expression on $CD3^+$, $CD3^+CD4^+$ and $CD3^+CD8^+$ T cells. HC=healthy controls, SLE=all enrolled SLE patients, SLE subgroup=defined group of SLE patients with SLEDAI ≤2, receiving a treatment of no more than hydroxychloroquine/chloroquine, prednisone ≤ 5mg and no DMARDs/biologicals.

Supplementary Table 4. Patient's characteristics

	all	steroids (prednisone)			treatment				
		no steroids	*≤ 5mg*	*> 5mg*	*no DMARD no biological no HCQ/CQ*	*HCQ/CQ mono*	*DMARD (+/- HCQ/CQ)*	*biological (+/- HCQ/CQ)*	*DMARD + biological (+/- HCQ/CQ)*
**SLEDAI**									
median	2	2	4	6	0	2	4	3	2
range	0 - 23	0-18	0-23	0-16	0-4	0-6	0-23	0-8	0-12
**treatment**									
no DMARD/biological/HCQ/CQ	8	5	1	1					
HCQ/CQ mono	30	25	4	1					
DMARD (+/- HCQ/CQ)	29	14	9	6					
biological (+/- HCQ/CQ)	5	2	3	1					
DMARD + biological (+/- HCQ/CQ)	13	3	9	2					
**steroids (prednisone)**									
no steroids	49				5	25	14	1	3
≤ 5mg	25				1	4	9	3	8
> 5mg	11				2	1	6	1	2

	all	SLEDAI			
		**0**	**1 - 4**	**5 - 8**	**> 8**
**treatment**					
**no DMARD/biological/HCQ/CQ**	8	5	3	0	0
**HCQ/CQ mono**	31	10	17	3	0
**DMARD (+/- HCQ/CQ)**	29	10	11	3	5
**biological (+/- HCQ/CQ)**	5	1	3	1	0
**DMARD + biological (+/- HCQ/CQ)**	13	4	7	1	2
**steroids (prednisone)**					
no steroids	50	8	18	6	5
**≤ 5mg**	25	6	16	2	2
**> 5mg**	11	2	2	4	3

Tabular list of the different treatments of all SLE patients enrolled (above). Indicated is also the median SLEDAI in relation to the different treatment groups. Indicated are the different treatments within groups of patients with a SLEDAI of 0, 1-4, 5-8 or >8 (below).

Supplementary Table 5. Immune status in NZB/WF1 animals treated with SCFA

	SCFA	untreated	
**spleen**	mean +/- SEM	mean +/- SEM	*p-value*
CD11b$^+$Ly6G^{hi} neutrophils/live cells	0.1674	0.1588	*ns*
	0.02529	0.02049	
CD11c^{hi} cDC/ live cells	0.4175	0.3323	*ns*
	0.05334	0.04009	
PDCA1^{hi} pDC/ live cells	0.4753	0.4566	*ns*
	0.03136	0.0275	
Ly6G$^-$CD11b$^+$ monocytic cells/ live cells	1.148	0.9448	*ns*
	0.1718	0.134	
TCRβ$^+$CD4$^+$ T cells/live cells	21.08	23.04	*ns*
	0.8633	0.8088	
CD44^{hi}/CD4$^+$ T cells	39.09	33.32	*ns*
	3.408	2.173	
CXCR5^{hi}PD1^{hi}/ CD4$^+$ T cells (T_{FH})	3.353	2.584	*ns*
	0.519	0.4024	
IFNγ$^+$/CD4$^+$ T cells	9.533	7.104	*ns*
	1.662	0.8536	
IL-17$^+$/CD4$^+$ T cells	0.116	0.1285	*ns*
	0.01262	0.01435	
IL-10$^+$/CD4$^+$ T cells	4.941	3.152	*ns*
	0.7694	0.3412	
FoxP3$^+$ /CD4$^+$ T cells (T_{reg})	24.09	20.57	*0.1698*
	1.325	0.9151	
CD103$^+$/FoxP3$^+$CD4$^+$ T cells	31.81	26.06	*0.0234*
(effector T_{reg})	1.588	0.9389	
TCRβ$^+$CD8$^+$ T cells/live cells	10.3	11.51	*ns*
	0.7499	0.7722	
CD44^{hi}/CD8$^+$ T cells	16.21	14.13	*ns*
	1.126	0.7025	
IFNγ$^+$/CD8$^+$ T cells	9.476	7.533	*ns*
	1.528	1.054	
TCRβ$^-$B220$^+$ B cells/live cells	44.226921	42.471733	*ns*
	1.4951014	1.2215557	
Fas^{hi}GL7^{hi}/B cells	2.736	2.246	*ns*
	0.3123	0.1868	
CD21^{lo}CD23^{hi} (follicular)/B cells	55.64	54.09	*ns*
	1.961	1.161	
CD21^{hi}CD23^{lo} (MZ)/B cells	18.09	21.09	*ns*
	1.621	1.115	
LC$^+$ CD138^{hi}/live cells	2.976	2.736	*ns*
	0.3941	0.233	
**kidney**			
CD45$^+$/live cells	11.73	10.91	*ns*
	1.227	1.002	
CD11b$^+$Ly6G^{hi} neutrophils/CD45$^+$ cells	1.046	1.014	*ns*
	0.1392	0.1299	
CD11c^{hi} cDC/CD45$^+$ cells	0.9627	1.077	*ns*
	0.1156	0.1518	
PDCA1^{hi} pDC/CD45$^+$ cells	0.2533	0.3589	*0.1620*
	0.0258	0.04507	
CD11b$^+$ monocytic cells/CD45$^+$ cells	7.164	6.24	*ns*
	0.826	0.5791	
TCRβ$^+$CD4$^+$ T cells/live cells	40.03	42.12	*ns*
	1.074	1.621	
CD44^{hi}/CD4$^+$ T cells	53.23	47.22	*ns*
	4.275	2.871	
IFNγ$^+$/CD4$^+$ T cells	7.985	8.467	*ns*
	2.119	1.636	
FoxP3$^+$/CD4$^+$ T cells (T_{reg})	17.23	17.11	*ns*
	0.9731	0.8044	
CD103$^+$FoxP3$^+$/CD4$^+$ T cells	25.61	17.31	*0.0041*
(effector T_{reg})	1.495	1.511	
TCRβ$^+$CD8$^+$ T cells/CD45$^+$ cells	16.89	16.19	*ns*
	0.7899	0.5346	
CD44^{hi}/CD8$^+$ T cells	44.19	36.23	*ns*
	5.308	3.027	
IFNγ$^+$/CD8$^+$ T cells	7.467	8.34	*ns*
	1.264	1.188	
TCRβ$^-$B220$^+$ B cells/CD45$^+$ cells	18.21	15.89	*ns*
	1.731	1.147	

Broad immune status evaluation in spleen and kidney of 28w old NZB/WF1 mice, receiving a SCFA-mix or untreated drinking water (n SCFA=10-18 mice and n untreated=10-19 mice). Results are expressed as mean +/- SEM; $p<0.05$ was considered significant, $p>0.2$ is indicated as *ns, not significant*.
